# Model misspecification and bias for inverse probability weighting estimators of average causal effects

**DOI:** 10.1002/bimj.202100118

**Published:** 2022-08-31

**Authors:** Ingeborg Waernbaum, Laura Pazzagli

**Affiliations:** ^1^ Department of Statistics Uppsala University Sweden and Institute for Evaluation of Labour Market and Education Policy IFAU Uppsala Sweden; ^2^ Centre for Pharmacoepidemiology Department of Medicine Solna Karolinska Institutet Stockholm Sweden

**Keywords:** average causal effects, comparing biases, outcome model, propensity score

## Abstract

Commonly used semiparametric estimators of causal effects specify parametric models for the propensity score (PS) and the conditional outcome. An example is an augmented inverse probability weighting (IPW) estimator, frequently referred to as a doubly robust estimator, because it is consistent if at least one of the two models is correctly specified. However, in many observational studies, the role of the parametric models is often not to provide a representation of the data‐generating process but rather to facilitate the adjustment for confounding, making the assumption of at least one true model unlikely to hold. In this paper, we propose a crude analytical approach to study the large‐sample bias of estimators when the models are assumed to be approximations of the data‐generating process, namely, when all models are misspecified. We apply our approach to three prototypical estimators of the average causal effect, two IPW estimators, using a misspecified PS model, and an augmented IPW (AIPW) estimator, using misspecified models for the outcome regression (OR) and the PS. For the two IPW estimators, we show that normalization, in addition to having a smaller variance, also offers some protection against bias due to model misspecification. To analyze the question of when the use of two misspecified models is better than one we derive necessary and sufficient conditions for when the AIPW estimator has a smaller bias than a simple IPW estimator and when it has a smaller bias than an IPW estimator with normalized weights. If the misspecification of the outcome model is moderate, the comparisons of the biases of the IPW and AIPW estimators show that the AIPW estimator has a smaller bias than the IPW estimators. However, all biases include a scaling with the PS‐model error and we suggest caution in modeling the PS whenever such a model is involved. For numerical and finite sample illustrations, we include three simulation studies and corresponding approximations of the large‐sample biases. In a dataset from the National Health and Nutrition Examination Survey, we estimate the effect of smoking on blood lead levels.

## 1 INTRODUCTION

Identifying an average causal effect of a treatment with observational data requires adjustment for background variables that affect both the treatment and the outcome under study. Often parametric models are assumed for parts of the joint distribution of the treatment, outcome, and background variables (covariates) and large‐sample properties of estimators are derived under the assumption that the parametric models are correctly specified.

Inverse probability weighting (IPW) estimators use the difference between the weighted means of the outcomes for the treatment groups as an estimator of the average causal effect. See, for example, the early paper by Hirano et al. (2003) for a nonparametric implementation of standard IPW estimators of the average causal effect. Under an assumption of no unmeasured confounding, IPW estimators reweight the observed outcomes to represent a full sample of potential outcomes, missing and observed, by letting each observed outcome account for itself and other individuals with similar characteristics. IPW estimators can be found in applied literature (see Chang et al., 2017; Kwon et al., 2015 for examples) and their properties have been generalized by Robins, Rotnitzky, and others (Robins & Rotnitzky, 1994; Robins et al., 1995, 2000) to address both confounding bias in observational studies and bias due to missing data. Vansteelandt et al. (2010) and Seaman and White (2013) provide reviews.

Properties of IPW estimators for estimating the average causal effect under the assumption that a parametric propensity score (PS) model is correctly specified have been described (il Kim et al., 2019; Lunceford & Davidian, 2004; Yao et al., 2010). Other studies of IPW estimators includes investigating properties when using different weights, for example, stabilized (Hernán et al., 2000; Hernán & Robins, 2006), normalized (Busso et al., 2014; Hirano & Imbens, 2001), or trimmed (Ma & Wang, 2020).

To decrease reliance on the choice of a parametric model and increase the efficiency of *augmented IPW (AIPW)* estimators, Robins and Rotnitzky (1994) proposed including a combination of regression adjustment with weighting based on the PS. See also the review by Seaman and Vansteelandt (2018). The AIPW estimators are referred to as doubly robust (DR) estimators (Bang & Robins, 2005; Tsiatis, 2007) because they are consistent estimators of the average causal effect if either a model for the PS or the outcome regression (OR) model is correct (Scharfstein et al., 1999). The efficiency of different DR estimators is a key property and the variances of the estimators have been described under correct specification of at least one of the models (Cao et al., 2009; Tan, 2010). When both models are correct, the estimator reaches the semiparametric efficiency bound described in Robins and Rotnitzky (1994). The large‐sample properties of IPW estimators with standard, normalized and variance minimized weights, together with an AIPW estimator were studied and compared in Lunceford and Davidian (2004) under correct specification of the PS and OR models. Multiply robust estimators allow several candidate models for the PS and OR, respectively. The property of multiple robustness means that the estimators are consistent for the true average treatment effect if any one of the multiple models is correctly specified (Han & Wang, 2013).

There are few studies of doubly or multiply robust estimators under misspecification of both (all) the PS and the OR models. Kang and Schafer (2007) studied and compared the performance of an AIPW estimator for missing data under misspecification of both the PS and OR model. They concluded that many DR methods perform better than simple IPW. However, a regression‐based estimator under a misspecified model was not improved upon. The paper was commented on and the relevance of the results was discussed by several authors. See, for example, Tsiatis and Davidian (2007), Tan (2007), and Robins et al. (2007). In Waernbaum (2012), a matching estimator was compared to IPW and AIPW estimators under misspecification of both the PS and OR models. Here, a robustness class for the matching estimator under misspecification of the PS model was described. Formulated in the missing data framework, Tan (2010) evaluated several semiparametric estimators, including IPW and AIPW estimators. In the evaluation, additional criteria were proposed describing robustness classes of the estimators. Vermeulen and Vansteelandt (2015) proposed a bias‐reduced AIPW estimator that locally minimizes the squared first‐order asymptotic bias under misspecification of both working models. One of the difficulties in the estimation of PSs occurs when the treatment groups have substantially different covariate distributions resulting in some PSs being close to zero or one. This lack of overlap raises issues with respect to model specification. Parametric binary response models, such as the commonly used probit and logit models, are similar in the middle areas of their arguments. However, for probabilities closer to zero or one, they tend to differ more resulting in the specified parametric model being more influential. In Zhou et al. (2020), misspecification of the PS linked to limited overlap is investigated for causal effect estimators using balancing weights (Li et al., 2018). Comparing IPW estimators with and without trimming with overlap weights, matching weights, and entropy weights, they find in extensive simulations that the latter three methods outperform the former (IPW and trimmed IPW) with respect to bias, root‐mean‐squared error and coverage (Zhou et al., 2020).

In this paper, we describe two commonly used IPW estimators and a prototypical AIPW estimator of the average causal effect under the assumption that none of the working models is correctly specified. For this purpose, we study the difference between the probability limit of the estimator under model misspecification and the true average causal effect. The purpose of this definition of the bias is that the estimators under study converge to a well‐defined limit, that is not, however, necessarily consistent for the true average causal effect. We study the biases of the (A)IPW estimators and compare them under the same misspecification of the PS model. The three estimators contain an error involving the ratio of the true PS and the limiting misspecified PS; however, the error affects the estimators in different ways. As the biases for the three estimators can be in different directions, we describe sufficient and necessary conditions using inequalities involving the absolute value of the biases. For a simple and a normalized IPW estimator, we show that the normalization in general moderates the bias due to the PS model misspecification. Comparing the IPW estimators to the AIPW estimator, the biases provide a means to describe when two wrong models are better than one, which would normally be the case for a moderate misspecification of the outcome model. Three simulation studies are performed to investigate the biases for finite samples. The data‐generating processes and the misspecified models from the simulation designs are also used for numerical approximations of the large‐sample properties derived in the paper.

The paper proceeds as follows. Section 2 presents the model and theory together with the estimators and their properties when the working models are correctly specified. Section 3 presents a general approach and associated assumptions to study model misspecification. In Section 4, the generic biases are derived and comparisons between the estimators are performed. We present three simulation studies in Section 5 containing both finite sample properties of the estimators and numerical large‐sample approximations. We apply the estimators under study on an observational dataset in Section 6 where we evaluate the effect of smoking on blood lead levels, and thereafter we conclude with a discussion.

## 2 MODEL AND THEORY

The potential outcome framework defines a causal effect as a comparison of potential outcomes that would be observed under different treatments (Rubin, 1974). Let *X* be a vector of pretreatment variables, referred to as covariates, *T* a binary treatment, with realized value T=1 if treated and T=0 if control. Under SUTVA (Rubin, 1980), the causal effect of the treatment is defined as a contrast between two potential outcomes, for example, the difference, Y(1)−Y(0), where *Y*(1) is the potential outcome under treatment and *Y*(0) is the potential outcome under the control treatment. The observed outcome *Y* is assumed to be the potential outcome for each level of the treatment Y=TY(1)+(1−T)Y(0), so that the data vector that we observe is $\left(T_{i},X_{i},Y_{i}\right)$, where i=1,…,n are assumed independent and identically distributed copies. In the remainder of the paper, we will drop the subscript *i* for the random variables when not needed. Since each individual only can be subject to one treatment, either *Y*(1) or *Y*(0) will be missing. If the treatment is randomized, the difference between the sample averages of the treated and controls will be an unbiased estimator of the average causal effect Δ=E[Y(1)−Y(0)], the parameter of interest. In the following, we will use the notation $\mu_{1}=E\left[Y\left(1\right)\right]$, $\mu_{0}=E\left[Y\left(0\right)\right]$ for the marginal expectations and $\mu_{1}\left(X\right)=E\left(Y\left(1\right)|X\right)$, $\mu_{0}\left(X\right)=E\left(Y\left(1\right)|X\right)$ for their conditional counterparts. We denote the probability of being treated conditional on the covariates, the PS by e(X)=P(T=1|X). When the treatment is not assigned randomly,common identification criteria include assumptions of no unmeasured confounding and overlap:

Assumption 1: (No unmeasured confounding) Y(t)⊥⊥T|X,t=0,1,

Assumption 2: (Overlap) η<P(T=1|X=x)<1−η, ∀x and some η>0,

where the assumption that P(T=1|X=x) is bounded away from zero and one guarantees the existence of a consistent estimator (Khan & Tamer, 2010). Under Assumptions 1 and 2, we can estimate the average causal effect by weighting the observed outcomes with the inverse of the PSs, because

$$\Delta =E\left[\frac{\mathrm{TY}}{e(X)}-\frac{(1-T)Y}{1-e(X)}\right],$$

leading to an estimator ${\hat{\Delta }}_{{\text{IPW}}_{1}}$ defined by:

(1) $${\hat{\Delta }}_{{\text{IPW}}_{1}}=\frac{1}{n}\sum_{i=1}^{n}\frac{T_{i}Y_{i}}{\hat{e}\left(X_{i}\right)}-\frac{1}{n}\sum_{i=1}^{n}\frac{\left(1-T_{i}\right)Y_{i}}{1-\hat{e}\left(X_{i}\right)}.$$

A common version of the simple IPW estimator in (1) is an IPW estimator ${\hat{\Delta }}_{{\text{IPW}}_{2}}$ with normalized weights

(2) $${\hat{\Delta }}_{{\text{IPW}}_{2}}={\left(\sum_{i=1}^{n}\frac{T_{i}}{\hat{e}\left(X_{i}\right)}\right)}^{-1}\sum_{i=1}^{n}\frac{T_{i}Y_{i}}{\hat{e}\left(X_{i}\right)}-{\left(\sum_{i=1}^{n}\frac{1-T_{i}}{1-\hat{e}\left(X_{i}\right)}\right)}^{-1}\sum_{i=1}^{n}\frac{\left(1-T_{i}\right)Y_{i}}{1-\hat{e}\left(X_{i}\right)}.$$

Using parametric IPW, we assume a finite‐dimensional model for $e\left(X\right)=e\left(X,\beta \right),\beta \in R^{p}$.

Assumption 3: (PS model) The PS e(X) follows a model e(X,β) parameterized by, $\beta =(\beta_{1},\text{…},\beta_{p})$, and $\hat{e}\left(X\right)$ is the estimated PS $e(X,\hat{\beta })$, with $\hat{\beta }$ a consistent estimator of β.

Under Assumptions 1–3, the IPW estimators are consistent estimators of the average causal effect Δ with asymptotic distribution $\sqrt{n}\left({\hat{\Delta }}_{{\text{IPW}}_{k}}-\Delta \right)∼N\left(0,\sigma^{2}{\text{IPW}}_{k}\right)$, k=1,2. Asymptotic properties of (1) and (2) are described in Lunceford and Davidian (2004) under an assumption of a logistic regression model for the treatment assignment.

Similar to the modeling of the PS, it can be assumed that the OR follows a parametric model $\mu_{t}\left(X\right)=\mu_{t}\left(X,\alpha_{t}\right),\alpha_{t}\in R^{p}$, t=0,1.

Assumption 4: (OR model) The conditional expectation, $\mu_{t}\left(X\right)=E\left(Y\left(t\right)|X\right)$, t=0,1, follows a model $\mu_{t}\left(X,\alpha_{t}\right)$, t=0,1 parameterized by $\alpha_{t}=\left(\alpha_{t1},\text{…},\alpha_{tq_{t}}\right)$, and ${\hat{\mu }}_{t}\left(X\right)$ is the estimated OR $\mu_{t}\left(X,{\hat{\alpha }}_{t}\right)$, with ${\hat{\alpha }}_{t}$ a consistent estimator of $\alpha_{t}$.

In addition, we study a prototypical AIPW estimator (Lunceford & Davidian, 2004; Tsiatis, 2007)

(3) $$\begin{array}{cc} {\hat{\Delta }}_{\text{AIPW}}= & \frac{1}{n}\sum_{i=1}^{n}\frac{T_{i}Y_{i}-\left(T_{i}-\hat{e}\left(X_{i}\right)\right){\hat{\mu }}_{1}\left(X_{i}\right)}{\hat{e}\left(X_{i}\right)}\\ - & \frac{1}{n}\sum_{i=1}^{n}\frac{\left(1-T_{i}\right)Y_{i}+\left(T_{i}-\hat{e}\left(X_{i}\right)\right){\hat{\mu }}_{0}\left(X_{i}\right)}{1-\hat{e}\left(X_{i}\right)}. \end{array}$$

Under Assumptions 1–4 and regularity conditions, the large‐sample distribution is $\sqrt{n}\left({\hat{\Delta }}_{\text{AIPW}}-\Delta \right)∼N\left(0,\sigma_{\text{AIPW}}^{2}\right)$.

For example, a model assumption for the treatment assignment could be a logistic regression model $e\left(X,\beta \right)={\left[1+\exp\left(-X^{\prime }\beta \right)\right]}^{-1}$ with $\hat{e}\left(X\right)$ the fitted values of the PS when $\hat{\beta }$ is a maximum likelihood estimator of β. The OR model could be a linear model $\mu_{t}\left(X,\alpha_{t}\right)=X^{\prime }\alpha_{t}$ where ${\hat{\mu }}_{t}\left(X\right)$, t=0,1 are the fitted values when ${\hat{\alpha }}_{t}$ is the ordinary least squares estimator.

## 3 MODEL MISSPECIFICATION

Our interest lies in the behaviors of the estimators when the PS and the OR models are misspecified. For this purpose, we replace Assumptions 3 and 4 with two other assumptions defining the probability limit of the estimators under a general misspecification. The misspecifications will further be used to define a general bias of the IPW and DR estimators. When the PS is misspecified an estimator, for example, a quasi‐maximum likelihood estimator (QMLE) is not consistent for β in Assumption 3. However, a probability limit for an estimator under model misspecification exists under general conditions, see, for example, White (1982, Theorem 2.2) for QMLE or Wooldridge (2010, Section 12.1) and Boos and Stefanski (2013, Theorem 7.1) for estimators that can be written as a solution of an estimating equation (M‐estimators).

In the following, and as an alternative to Assumptions 1 and 4, we will assume that such limits exist. Below we define an estimator ${\hat{e}}^{*}\left(X\right)$ of the PS under a misspecified model $e(X,\beta^{*})$.

Assumption 5 (Misspecified PS‐model parameters) Let ${\hat{\beta }}^{*}$ be an estimator under model misspecification, $e(X,\beta^{*})$, then ${\hat{\beta }}^{*}\overset{p}{⟶}\beta^{*}$.

Under model misspecification, the probability limit of $\hat{\beta^{*}}$ is generally well defined; however, $e(X,\beta^{*})$ is not equal to the true PS e(X). In the following, we use the notation ${\hat{e}}^{*}\left(X\right)$ as the estimated PS under model misspecification and $e^{*}\left(X\right)=e\left(X,\beta^{*}\right)$ under Assumption 5. Below we give an example for true and misspecified parametric models, however, for Assumption 5, we do not need the existence of a true parametric model. We use the concept of quasi‐maximum likelihood used for maximum likelihood estimators when parts of the distribution are misspecified.

Example 1 For one confounder *X* and a true PS model, $e\left(X,\beta \right)={\left[1+\exp\left(-\beta_{0}-\beta_{1}X-\beta_{2}X^{2}\right)\right]}^{-1}$ assume that we misspecify the PS with a probit model $e\left(X,\beta^{*}\right)=\Psi \left(-\beta_{0}^{*}-\beta_{1}^{*}X\right)$, that is, we misspecify the link function and omit a second‐order term. Let ${\hat{\beta }}^{*}=\left({\hat{\beta }}_{0}^{*},{\hat{\beta }}_{1}^{*}\right)$ be the QML estimator of the parameters in $e(X,\beta^{*})$ obtained by maximizing the quasi‐likelihood

$$\ln L=\sum_{i=1}^{n}\left(T_{i}\ln e\left(X_{i},\beta^{*}\right)+\left(1-T_{i}\right)\ln\left(1-e\left(X_{i},\beta^{*}\right)\right)\right).$$

Then ${\hat{e}}^{*}\left(X\right)=\Psi \left(-{\hat{\beta }}_{0}^{*}-{\hat{\beta }}_{1}^{*}X\right)$, ${\hat{\beta }}^{*}=\left({\hat{\beta }}_{0}^{*},{\hat{\beta }}_{1}^{*}\right)\overset{p}{⟶}\beta^{*}=\left(\beta_{0}^{*},\beta_{1}^{*}\right)$ under Assumption 5 and $e^{*}\left(X\right)=\Psi \left(-\beta_{0}^{*}-\beta_{1}^{*}X\right)$, where Ψ() is the CDF of a standard normal random variable.

When considering the existence of true and misspecified parametric models, as illustrated in Example 1, the parameters in β and the limiting parameters $\beta^{*}$ under the misspecified model need not to be of the same dimension. For instance, the true model could contain higher order terms and interactions that are not present in the estimation model.

The next assumption concerns overlap under model misspecification.

Assumption 6: (Overlap under misspecification) $\nu <e^{*}\left(X\right)<1-\nu$, for some ν>0.

In addition to the PS model, we also consider misspecified OR models, $\mu_{t}\left(X,\alpha_{t}^{*}\right)$, t=0,1. Denote by ${\hat{\alpha }}_{t}^{*}$, t=0,1 the estimator of the parameters in $\mu_{t}\left(X,\alpha_{t}^{*}\right)$.

Assumption 7: (Misspecified OR model parameters) Let ${\hat{\alpha }}_{t}^{*}$ be an estimator under model misspecification $\mu_{t}\left(X,\alpha_{t}^{*}\right)$, t=0,1, then ${\hat{\alpha }}_{t}^{*}\overset{p}{⟶}\alpha_{t}^{*}$, t=0,1.

In the following, we use the notation ${\hat{\mu }}_{t}^{*}\left(X\right)$ as the estimated OR under model misspecification and $\mu_{t}^{*}\left(X\right)=\mu_{t}\left(X,\alpha_{t}^{*}\right)$ under Assumption 7 and $\mu_{t}^{*}$ for the expected value $E[\mu_{t}^{*}\left(X\right)]$, t=0,1.

Assumptions 5 and 7 are defined for misspecified PS and OR models for the purpose of describing their influence on the estimation of Δ. The estimators (1)–(3) can be written by estimating equations where the equations solving for the PS and OR parameters are set up below the main equation for the (A)IPW estimators. See, for example, Lunceford and Davidian (2004) and Williamson et al. (2014). Assuming parametric PS and OR models, the IPW estimators correspond to solving 2+p estimating equations $\sum_{i=1}^{n}\psi \left(\theta ,Y_{i},T_{i},X_{i}\right)=0$ for the parameters $\theta_{{\text{IPW}}_{k}}=\left(\mu_{1},\mu_{0},\beta \right)$, k=1,2, and for the AIPW estimator $2+p+q_{1}+q_{0}$ estimating equations for the parameters $\theta_{\text{AIPW}}=\left(\mu_{1},\mu_{0},\beta ,\alpha_{1},\alpha_{0}\right)$. Using the notation for the misspecified models in Assumptions 5 and 7, the estimating equations change according to the dimensions of the parameters $\beta^{*}$ and $\alpha_{t}^{*}$, t=0,1. A key condition for Assumptions 5 and 7 to hold is that the misspecification of the PS and/or OR provides estimating equations that uniquely define the parameters $\beta^{*}$ and $\alpha_{t}^{*}$, t=0,1, although, as a consequence of the misspecification, the resulting (A)IPW estimators will be biased. In the next section, we present the asymptotic bias for the (A)IPW estimators under study with general expressions including the limits of the misspecified PS and OR models.

## 4 BIAS RESULTING FROM MODEL MISSPECIFICATION

### 4.1 General biases

We study the large‐sample bias of ${\hat{\Delta }}_{{\text{IPW}}_{1}}$, ${\hat{\Delta }}_{{\text{IPW}}_{2}}$, and ${\hat{\Delta }}_{\text{AIPW}}$ under model misspecification and define the estimators ${\hat{\Delta }}_{{\text{IPW}}_{1}}^{*}$, ${\hat{\Delta }}_{{\text{IPW}}_{2}}^{*}$, and ${\hat{\Delta }}_{\text{AIPW}}^{*}$ by replacing $\hat{e}\left(X\right)$ in Equations (1)–(3) with ${\hat{e}}^{*}\left(X\right)$. For the AIPW estimator, we additionally replace ${\hat{\mu }}_{t}\left(X\right)$ with ${\hat{\mu }}_{t}^{*}\left(X\right)$, t=0,1.

To assess the properties of the estimators, we assume 1, 2, 5, 6, and 7 and regularity conditions (see Appendix A.1). We evaluate the difference between the probability limits of the estimators under model misspecification and the average causal effect Δ for the (A)IPW estimators.

Under Assumptions 1–2 and 5–6, the biases under model misspecification for ${\hat{\Delta }}_{{\text{IPW}}_{1}}^{*}$ and ${\hat{\Delta }}_{{\text{IPW}}_{2}}^{*}$ are

(4) $${\hat{\Delta }}_{{\text{IPW}}_{1}}^{*}-\Delta \overset{p}{⟶}E\left[\frac{e(X)}{e^{*}\left(X\right)}\mu_{1}\left(X\right)\right]-E\left[\frac{1-e(X)}{1-e^{*}\left(X\right)}\mu_{0}\left(X\right)\right]-\left(\mu_{1}-\mu_{0}\right),$$

(5) $${\hat{\Delta }}_{{\text{IPW}}_{2}}^{*}-\Delta \overset{p}{⟶}\frac{E\left[\frac{e(X)}{e^{*}\left(X\right)}\mu_{1}\left(X\right)\right]}{E\left[\frac{e(X)}{e^{*}\left(X\right)}\right]}-\frac{E\left[\frac{1-e(X)}{1-e^{*}\left(X\right)}\mu_{0}\left(X\right)\right]}{E\left[\frac{1-e(X)}{1-e^{*}\left(X\right)}\right]}-\left(\mu_{1}-\mu_{0}\right).$$

Under Assumptions 1–2 and 5–7, the bias under model misspecification for ${\hat{\Delta }}_{\text{AIPW}}^{*}$ is

(6) $$\begin{array}{cc} {\hat{\Delta }}_{\text{AIPW}}^{*}-\Delta \overset{p}{⟶} & E\left[\frac{\left(e\left(X\right)-e^{*}\left(X\right)\right)\left(\mu_{1}\left(X\right)-\mu_{1}^{*}\left(X\right)\right)}{e^{*}\left(X\right)}\right]\\ & +E\left[\frac{\left[e\left(X\right)-e^{*}\left(X\right)\right]\left(\mu_{0}\left(X\right)-\mu_{0}^{*}\left(X\right)\right)}{\left(1-e^{*}\left(X\right)\right)}\right]. \end{array}$$

We refer to Equations (4)–(6) as the biases of the respective estimators, that is, Bias$\left({\hat{\Delta }}_{{\text{IPW}}_{1}}^{*}\right)$, Bias$\left({\hat{\Delta }}_{{\text{IPW}}_{2}}^{*}\right)$, and Bias$\left({\hat{\Delta }}_{\text{AIPW}}^{*}\right)$ although they are the difference between the probability limits of the estimators and the true Δ and not the difference in expectations. The double robustness property of ${\hat{\Delta }}_{\text{AIPW}}^{*}$ is displayed by Equation (6) because if either $e\left(X\right)=e^{*}\left(X\right)$ or $\mu_{t}\left(X\right)=\mu_{t}^{*}\left(X\right)$, t=0,1, we have that ${\hat{\Delta }}_{\text{AIPW}}^{*}\overset{p}{⟶}\Delta$.

### 4.2 Comparisons

The consequences of model misspecification for the estimators, respectively, can further be investigated from the general biases in Equations (4)–(6).

To study the role of the model misspecification, we compare the biases in Section 4.1 for two separate parts of the estimator. The first part concerns the bias with respect to μ_1_ and the second part with respect to μ_0_. The motivation behind this component‐wise comparison is that if each of the estimators of $\mu_{1},\mu_{0}$ is unbiased, then the resulting estimator of $\Delta =\mu_{1}-\mu_{0}$ is also unbiased. The inverse relationship between the model errors $e\left(X\right)/e^{*}\left(X\right)$ and $\left(1-e\left(X\right)\right)/\left(1-e^{*}\left(X\right)\right)$ has the result that the contribution to the overall bias from μ_1_ and μ_0_ may, in general, be of the same sign (see Appendix A.3). We define ${\text{Bias}}_{1}\left({\hat{\Delta }}_{{\text{IPW}}_{1}}^{*}\right)$, ${\text{Bias}}_{1}\left({\hat{\Delta }}_{{\text{IPW}}_{2}}^{*}\right)$, and ${\text{Bias}}_{1}\left({\hat{\Delta }}_{\text{AIPW}}^{*}\right)$ as

(7) $$\begin{array}{cc} & {\text{Bias}}_{1}\left({\hat{\Delta }}_{{\text{IPW}}_{1}}^{*}\right)=E\left[\frac{e(X)}{e^{*}\left(X\right)}\mu_{1}\left(X\right)\right]-\mu_{1}, \end{array}$$

(8) $$\begin{array}{cc} & {\text{Bias}}_{1}\left({\hat{\Delta }}_{{\text{IPW}}_{2}}^{*}\right)=\frac{E\left[\frac{e(X)}{e^{*}\left(X\right)}\mu_{1}\left(X\right)\right]}{E\left[\frac{e(X)}{e^{*}\left(X\right)}\right]}-\mu_{1}, \end{array}$$

(9) $$\begin{array}{cc} & {\text{Bias}}_{1}\left({\hat{\Delta }}_{\text{AIPW}}^{*}\right)=E\left[\left(\frac{e(X)}{e^{*}\left(X\right)}-1\right)\left(\mu_{1}\left(X\right)-\mu_{1}^{*}\left(X\right)\right)\right]. \end{array}$$

For ${\hat{\Delta }}_{{\text{IPW}}_{1}}^{*}$, the expression in (7) shows that the bias consists of a scaling between the model error $e\left(X\right)/e^{*}\left(X\right)$ and the conditional outcome $\mu_{1}\left(X\right)$. If the distribution of $e\left(X\right)/e^{*}\left(X\right)$ is positively skewed resulting in over estimation of the weighted mean $E[\frac{e(X)}{e^{*}\left(X\right)}\mu_{1}\left(X\right)]$, we see that for ${\hat{\Delta }}_{{\text{IPW}}_{2}}^{*}$, the bias is mitigated because $E[e\left(X\right)/e^{*}\left(X\right)]>1$. A similar effect is obtained for a negatively skewed distribution of $e\left(X\right)/e^{*}\left(X\right)$ where $E[e\left(X\right)/e^{*}\left(X\right)]<1$. There is no correspondence to this bias reduction for ${\text{Bias}}_{1}\left({\hat{\Delta }}_{{\text{IPW}}_{1}}^{*}\right)$.

The sign of the two biases in (7) and in (8) depends on the covariance of the PS‐model errors and the conditional outcome $\text{cov}[\frac{e(X)}{e^{*}\left(X\right)},\mu_{1}\left(X\right)]$, because

$$\begin{array}{c} {\text{Bias}}_{1}\left({\hat{\Delta }}_{{\text{IPW}}_{1}}^{*}\right)=\text{cov}\left[\frac{e(X)}{e^{*}\left(X\right)},\mu_{1}\left(X\right)\right]+E\left[\frac{e(X)}{e^{*}\left(X\right)}-1\right]\mu_{1}, \end{array}$$

and

$$\begin{array}{c} {\text{Bias}}_{1}\left({\hat{\Delta }}_{{\text{IPW}}_{2}}^{*}\right)=\frac{\text{cov}\left[\frac{e(X)}{e^{*}\left(X\right)},\mu_{1}\left(X\right)\right]}{E\left[\frac{e(X)}{e^{*}\left(X\right)}\right]}, \end{array}$$

implying that the biases can be in different directions for the same model misspecification. Here, ${\text{Bias}}_{1}\left({\hat{\Delta }}_{{\text{IPW}}_{2}}^{*}\right)$ depends only on the sign of the covariance, $\text{cov}[\frac{e(X)}{e^{*}\left(X\right)},\mu_{1}\left(X\right)]$, whereas this is not the case for ${\text{Bias}}_{1}\left({\hat{\Delta }}_{{\text{IPW}}_{1}}^{*}\right)$. It is not surprising that the covariance of $e\left(X\right)/e^{*}\left(X\right)$ and $\mu_{1}\left(X\right)$ plays a role for the bias of the estimators. If $\mu_{1}\left(X\right)$ was a constant, it could be taken out of the expectations of the first terms in (7) and (8) and the PS‐model ratio, $e\left(X\right)/e^{*}\left(X\right)$, would be canceled by the denominator $E[e\left(X\right)/e^{*}\left(X\right)]$ in (8). In this case, the bias for ${\hat{\Delta }}_{{\text{IPW}}_{2}}^{*}$ would be 0.

Next, we investigate inequalities involving the absolute values of the biases in Equations (7)–(9). (All derivations are given in Appendix A.3). The results can be directly applied for μ_0_ by replacing $e\left(X\right)/e^{*}\left(X\right)$, with $\left(1-e\left(X\right)\right)/\left(1-e^{*}\left(X\right)\right)$ and $\mu_{1}\left(X\right)$ with $\mu_{0}\left(X\right)$, see Appendix A.3.

First, to study the role of normalization for the IPW estimators, we compare ${\text{Bias}}_{1}\left({\hat{\Delta }}_{{\text{IPW}}_{1}}^{*}\right)$ and ${\text{Bias}}_{1}\left({\hat{\Delta }}_{{\text{IPW}}_{2}}^{*}\right)$.

A sufficient and necessary condition for

(10) $$\left|{\text{Bias}}_{1}\left({\hat{\Delta }}_{{\text{IPW}}_{2}}^{*}\right)\right|<\left|{\text{Bias}}_{1}\left({\hat{\Delta }}_{{\text{IPW}}_{1}}^{*}\right)\right|,$$

is that

(11) $$\left|\text{cov}\left[\frac{e(X)}{e^{*}\left(X\right)},\mu_{1}\left(X\right)\right]\right|<\left|E\left[\frac{e(X)}{e^{*}\left(X\right)}\right]E\left[\frac{e(X)}{e^{*}\left(X\right)}\mu_{1}\left(X\right)\right]-\mu_{1}\right|.$$

That is, the absolute value of the covariance between the PS‐model ratio and the conditional outcome is smaller than the absolute value of ${\text{Bias}}_{1}\left({\hat{\Delta }}_{{\text{IPW}}_{2}}^{*}\right)$ scaled with the PS‐model ratio.

To study the issue of misspecifying two models instead of one, we investigate the difference between the bias of the IPW estimators (7) and (8) and the bias of the AIPW estimator (9). We give a necessary condition for the bias of the AIPW estimator to be smaller than the bias of the simple IPW estimator:

If

$$\left|{\mathrm{Bias}}_{1}\left({\hat{\Delta }}_{\text{AIPW}}^{*}\right)\right|<\left|{\mathrm{Bias}}_{1}\left({\hat{\Delta }}_{{\text{IPW}}_{1}}^{*}\right)\right|,$$

then

(12) $$\left|E\left[\left(\frac{e(X)}{e^{*}\left(X\right)}-1\right)\mu_{1}^{*}\left(X\right)\right]\right|<2\cdot \left|E\left[\left(\frac{e(X)}{e^{*}\left(X\right)}-1\right)\mu_{1}\left(X\right)\right]\right|.$$

By (12), we see that if the AIPW estimator improves upon the simple IPW estimator under misspecification of both the PS and the OR model, then the absolute value of the misspecified outcome model is less than double the absolute value of the true conditional mean under the same scaling of the PS‐model error, $e\left(X\right)/e^{*}\left(X\right)-1$.

A sufficient condition for the AIPW estimator to have a smaller bias than the simple IPW estimator can be expressed as a comparison between the misspecified OR model and the true conditional outcomes under the same PS‐model error.

If

(13) $$\begin{array}{c} \begin{array}{cc} & \left|E\left[\left(\frac{e(X)}{e^{*}\left(X\right)}-1\right)\mu_{1}^{*}\left(X\right)\right]\right|<2\cdot \left|E\left[\left(\frac{e(X)}{e^{*}\left(X\right)}-1\right)\mu_{1}\left(X\right)\right]\right|,\\ & \text{and}\;E\left[\left(\frac{e(X)}{e^{*}\left(X\right)}-1\right)\mu_{1}^{*}\left(X\right)\right]\;\text{and}\;E\left[\left(\frac{e(X)}{e^{*}\left(X\right)}-1\right)\mu_{1}\left(X\right)\right], \end{array} \end{array}$$

are either both positive or both negative, then $|{\mathrm{Bias}}_{1}\left({\hat{\Delta }}_{\text{AIPW}}^{*}\right)\left|<\right|{\mathrm{Bias}}_{1}\left({\hat{\Delta }}_{{\text{IPW}}_{1}}^{*}\right)|$.

To provide a numerical example, we assume a second‐order model in one variable and obtain the misspecified models' limits by omitting the second order term in both the PS (logistic regression) and the OR model (linear model). We use numerical approximations to provide values for the parameters in $e^{*}\left(X\right)$ and $\mu_{t}^{*}\left(X\right)$, t=0,1 under the given true and misspecified models.

Example 2 For a covariate X∼Uniform(−2,2) and a binary treatment T∼Bernoulli(e(X)), we assume a logistic PS model and a linear conditional outcome and misspecified nonlinearities in the models

$$\begin{array}{cc} & e\left(X\right)={\left[1+\exp[\left(1+0.5X-0.1X^{2}\right)\right]}^{-1},\;e^{*}\left(X\right)={\left[1+\exp(0.87+0.52X)\right]}^{-1},\\ & \mu_{1}\left(X\right)=3.5+X+0.7X^{2},\;\mu_{1}^{*}\left(X\right)=4.42+0.76X. \end{array}$$

In this example, we have that the inequality in (11) holds

$$\begin{array}{cc} & \text{cov}\left[\frac{e(X)}{e^{*}\left(X\right)},\mu_{1}\left(X\right)\right]=0.12,\\ \; & E\left[\frac{e(X)}{e^{*}\left(X\right)}\right]E\left[\frac{e(X)}{e^{*}\left(X\right)}\mu_{1}\left(X\right)\right]-\mu_{1}=0.16. \end{array}$$

We expect based on previous calculations that $|{\mathrm{Bias}}_{1}\left({\hat{\Delta }}_{{\text{IPW}}_{2}}^{*}\right)\left|<\right|{\mathrm{Bias}}_{1}\left({\hat{\Delta }}_{{\text{IPW}}_{1}}^{*}\right)|$. Here, we have that Bias${}_{1}\left({\hat{\Delta }}_{{\text{IPW}}_{1}}^{*}\right)=0.16$, Bias${}_{1}\left({\hat{\Delta }}_{{\text{IPW}}_{2}}^{*}\right)=0.12$, confirming the result. To compare Bias${}_{1}\left({\hat{\Delta }}_{{\text{IPW}}_{1}}^{*}\right)$ with Bias${}_{1}\left({\hat{\Delta }}_{\text{AIPW}}^{*}\right)$, we check to see that

$$\begin{array}{cc} & E\left[\left(\frac{e(X)}{e^{*}\left(X\right)}-1\right)\mu_{1}^{*}\left(X\right)\right]=0.07,\\ \; & E\left[\left(\frac{e(X)}{e^{*}\left(X\right)}-1\right)\mu_{1}\left(X\right)\right]=0.16, \end{array}$$

are both positive, which is consistent with the sufficient conditions for

$|{\text{Bias}}_{1}\left({\hat{\Delta }}_{\text{AIPW}}^{*}\right)\left|<\right|{\text{Bias}}_{1}\left({\hat{\Delta }}_{{\text{IPW}}_{1}}^{*}\right)|$ of Equation (13). To confirm, Bias${}_{1}\left({\hat{\Delta }}_{\text{AIPW}}^{*}\right)=0.09$ that is smaller than 0.16.

Comparing the biases between the AIPW estimator and the IPW estimator with normalized weights, we investigate the inequality $|{\mathrm{Bias}}_{1}\left({\hat{\Delta }}_{\text{AIPW}}^{*}\right)\left|<\right|{\mathrm{Bias}}_{1}\left({\hat{\Delta }}_{{\text{IPW}}_{2}}^{*}\right)|$. Here, we see that for $|{\mathrm{Bias}}_{1}\left({\hat{\Delta }}_{\text{AIPW}}^{*}\right)\left|<\right|{\mathrm{Bias}}_{1}\left({\hat{\Delta }}_{{\text{IPW}}_{2}}^{*}\right)|$ to be true, the misspecified OR model is included in the necessary condition:

(14) $$\begin{array}{cc} & E\left[\left(\frac{e(X)}{e^{*}\left(X\right)}-1\right)\left(\mu_{1}\left(X\right)\right)\right]-\frac{\left|\text{cov}\left[\frac{e(X)}{e^{*}\left(X\right)},\mu_{1}\left(X\right)\right]\right|}{E\left[\frac{e(X)}{e^{*}\left(X\right)}\right]}<E\left[\left(\frac{e(X)}{e^{*}\left(X\right)}-1\right)\mu_{1}^{*}\left(X\right)\right]\\ < & E\left(\left[\frac{e(X)}{e^{*}\left(X\right)}-1\right]\left[\mu_{1}\left(X\right)\right]\right)+\frac{\left|\text{cov}\left[\frac{e(X)}{e^{*}\left(X\right)},\mu_{1}\left(X\right)\right]\right|}{E\left[\frac{e(X)}{e^{*}\left(X\right)}\right]}. \end{array}$$

By (14), we see that in order for the AIPW estimator to improve upon the normalized IPW estimator, the (PS‐error scaled) outcome misspecification must lie within an interval defined by the true conditional outcome and the absolute value of the covariance of $e\left(X\right)/e^{*}\left(X\right)$ and $\mu_{1}\left(X\right)$. This means that the smaller the covariance, the greater the accuracy of the outcome model for ${\hat{\Delta }}_{\text{AIPW}}^{*}$ to be less biased than ${\hat{\Delta }}_{{\text{IPW}}_{2}}^{*}$.

For a sufficient condition for $|{\mathrm{Bias}}_{1}\left({\hat{\Delta }}_{\text{AIPW}}^{*}\right)\left|<\right|{\mathrm{Bias}}_{1}\left({\hat{\Delta }}_{{\text{IPW}}_{2}}^{*}\right)|$, we have that if

(15) $$\begin{array}{c} \begin{array}{cc} & E\left[\left(\frac{e(X)}{e^{*}\left(X\right)}-1\right)\mu_{1}\left(X\right)\right]\;\text{and}\;\text{cov}\;\left[\frac{e(X)}{e^{*}\left(X\right)},\mu_{1}\left(X\right)\right]\\ & \text{are}\;\text{either}\;\text{both}\;\text{positive}\;\text{or}\;\text{both}\;\text{negative,}\;\text{and}\\ & \left|E\left[\left(\frac{e(X)}{e^{*}\left(X\right)}-1\right)\mu_{1}^{*}\left(X\right)\right]\right|<\left|E\left[\left(\frac{e(X)}{e^{*}\left(X\right)}-1\right)\mu_{1}\left(X\right)\right]+\frac{\text{cov}\left[\frac{e(X)}{e^{*}\left(X\right)},\mu_{1}\left(X\right)\right]}{E\left[\frac{e(X)}{e^{*}\left(X\right)}\right]}\right|, \end{array} \end{array}$$

then

$$\left|E\left[\left(\frac{e(X)}{e^{*}\left(X\right)}-1\right)\left(\mu_{1}\left(X\right)-\mu_{1}^{*}\left(X\right)\right)\right]\right|<\left|\frac{E\left[\frac{e(X)}{e^{*}\left(X\right)}\mu_{1}\left(X\right)\right]}{E\left[\frac{e(X)}{e^{*}\left(X\right)}\right]}-\mu_{1}\right|.$$

Illustrating the sufficient conditions with the data‐generating process in Example 2

$$\begin{array}{c} E\left[\left(\frac{e(X)}{e^{*}\left(X\right)}-1\right)\mu_{1}\left(X\right)\right]=0.16\;,\;\text{cov}\left[\frac{e(X)}{e^{*}\left(X\right)},\mu_{1}\left(X\right)\right]=0.12 \end{array}$$

are both positive, and

$$\begin{array}{cc} \left|E\left[\left(\frac{e(X)}{e^{*}\left(X\right)}-1\right)\mu_{1}^{*}\left(X\right)\right]\right| & <\left|E\left[\left(\frac{e(X)}{e^{*}\left(X\right)}-1\right)\left(\mu_{1}\left(X\right)\right)\right]+\frac{\left|\text{cov}\left[\frac{e(X)}{e^{*}\left(X\right)},\mu_{1}\left(X\right)\right]\right|}{E\left[\frac{e(X)}{e^{*}\left(X\right)}\right]}\right|,\\ 0.07 & <0.28, \end{array}$$

in agreement with (15).

**FIGURE 1:**
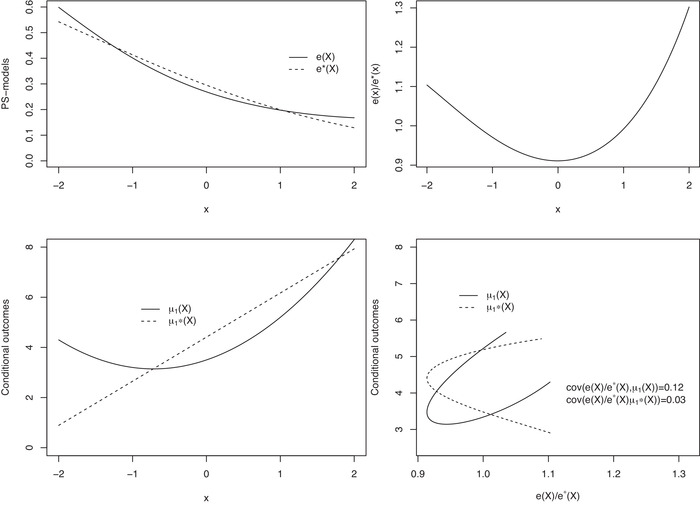
Illustration of the components of the biases using the data‐generating process from Example 2. Top left: e(X) and $e^{*}\left(X\right)$ by *X*; top right: $e\left(X\right)/e^{*}\left(X\right)$ by *X*; bottom left: $\mu_{1}\left(X\right)$ and $\mu_{1}^{*}\left(X\right)$ by *X*; and bottom right: $\mu_{1}\left(X\right)$ and $\mu_{1}^{*}\left(X\right)$ by $e\left(X\right)/e^{*}\left(X\right)$

Summarizing the results of the comparisons of the bias conditions, we note that the expected values of the product of the PS‐model error and the true and misspecified conditional outcomes play important roles. Here, the covariances of the PS‐model ratio and the true and misspecified conditional outcomes are two of their respective components. In Figure 1, we illustrate these parts with the data‐generating processes from Example 2. The PS‐model ratio deviates from 1 for both small and large values of *X*, but more so for larger values of *X*. Since both conditional outcomes $\mu_{1}\left(X\right)$ and $\mu_{1}^{*}\left(X\right)$ are strictly increasing, both covariances are positive ($\text{cov}[e\left(X\right)/e^{*}\left(X\right),\mu_{1}\left(X\right)]=0.12$ and $\text{cov}[e\left(X\right)/e^{*}\left(X\right),\mu_{1}^{*}\left(X\right)]=0.03$) owing to the PS‐model ratio being larger for larger values of *X*. The interval characterization of the described conditions implies that if the two covariances are of the same magnitude, the bias of ${\hat{\Delta }}_{\text{AIPW}}^{*}$ will often be smaller than the biases of ${\hat{\Delta }}_{{\text{IPW}}_{1}}^{*}$ and ${\hat{\Delta }}_{{\text{IPW}}_{2}}^{*}$.

## 5 SIMULATION STUDIES

### 5.1 Design

To investigate the asymptotic biases described in Section 4 and also the finite‐sample performance of ${\hat{\Delta }}_{{\text{IPW}}_{1}}^{*}$, ${\hat{\Delta }}_{{\text{IPW}}_{2}}^{*}$, and ${\hat{\Delta }}_{\text{AIPW}}^{*}$ under model misspecification, we perform three simulation studies with three different designs A–C. The first part of the simulations evaluates the finite‐sample performance of the estimators and consist of 1000 replications of sample sizes 500, 1000, and 5000. In addition to the simulation results, we also give numerical approximations to the asymptotic biases by fitting the misspecified models with a large sample N=1,000,000. The simulations and numerical approximations are carried out using R (R Core Team, 2020). The misspecified models are fitted with the glm() function and were selected in order to be well‐known simple models that could have been chosen in practice by a data analyst. The link functions together with the true parameter values are given in Tables 1 and 2, which also contain the details for the misspecified models.

**TABLE 1:** Simulation Designs A–C

	True model		MISSPECIFIED MODEL	
Models	Class	Linear predictor and parameter values	Class	Linear predictor
*Simulation 1*				
		$\beta =\left(-1,0.6,0.1,-0.9,0.1,0.7\right),\alpha_{0}=\left(3,0.5,0.2,0.5,0.2,0.2\right),\alpha_{1}=\left(4,1.1,0.1,0.5,0.3,0.2\right)$		
*Design A*				
PS	Binomial, logit	$1,X_{1},X_{2},X_{1}^{2},X_{2}^{2},X_{3}$	Binomial, logit	$1,X_{1},X_{2},X_{2}^{2},X_{3}$
OR	Gaussian, identity	$1,X_{1},X_{2},X_{1}^{2},X_{2}^{2},X_{3}$	Gaussian, identity	$1,X_{1},X_{2},X_{2}^{2},X_{3}$
*Design B*				
PS	Binomial, logit	$1,X_{1},X_{2},X_{1}^{2},X_{2}^{2},X_{3}$	Binomial, logit	$1,X_{1},X_{2},X_{3}$
OR	Gaussian, identity	$1,X_{1},X_{2},X_{1}^{2},X_{2}^{2},X_{3}$	Gaussian, identity	$1,X_{1},X_{2},X_{3}$
*Design C*				
PS	Binomial, cauchit	$1,X_{1},X_{2},X_{1}^{2},X_{2}^{2},X_{3}$	Binomial, logit	$1,X_{1},X_{2},X_{3}$
OR	Gamma, identity	$1,X_{1},X_{2},X_{1}^{2},X_{2}^{2},X_{3}$	Gaussian, identity	$1,X_{1},X_{2},X_{3}$
*Simulation 2*				
		$\beta =\left(-0.9,1,1.4,1,0.2,0.3\right),\alpha_{0}=\left(0,1,1,2,0.5,1\right),\alpha_{1}=\left(1.5,1.5,2,-0.8,0.9,0.4\right)$		
		$1,Z_{1}=X_{1}+X_{2}+X_{3},Z_{2}=X_{1}+X_{3}+X_{4}$,		
		$1,Z_{3}=X_{1}+X_{2},Z_{4}=X_{1}+X_{3}$		
*Design A*				
PS	Binomial, logit	$1,X_{1},X_{2},X_{1}^{2},X_{2}^{2},X_{3}$	Binomial, logit	$1,X_{1},X_{2},X_{3}$
OR	Gaussian, identity	$1,X_{1},X_{3},X_{1}^{2},X_{3}^{2},X_{4}$	Gaussian, identity	$1,X_{1},X_{3},X_{4}$
*Design B*				
PS	Binomial, logit	$1,X_{1},X_{2},X_{1}^{2},X_{2}^{2},X_{3}$	Binomial, logit	1, *Z*_1_
OR	Gaussian, identity	$1,X_{1},X_{3},X_{1}^{2},X_{3}^{2},X_{4}$	Gaussian, identity	1, *Z*_2_
*Design C*				
PS	Binomial, logit	$1,X_{1},X_{2},X_{1}^{2},X_{2}^{2},X_{3}$	Binomial, logit	1, *Z*_3_
OR	Gaussian, identity	$1,X_{1},X_{3},X_{1}^{2},X_{3}^{2},X_{4}$	Gaussian, identity	1, *Z*_4_

**TABLE 2:** Simulation Designs A–C

	True model		MISSPECIFIED MODEL	
Models	Class	Linear predictor and parameter values	Class	Linear predictor
*Simulation 3*				
		$\beta =\left(-0.20,0.30,0.15,0.22,0.15,-0.15\right),\alpha_{0}=\left(9,-0.05,0.05,-0.05\right),\alpha_{1}=\left(10,-0.05,0.05,-0.05\right)$		
		$1,M_{1}=\exp\left(0.10X_{1}\right),M_{2}=X_{2}\left(1+X_{1}\right)+10,M_{3}={\left(0.04X_{3}+0.60\right)}^{2},M_{4}={\left(X_{4}+20\right)}^{2}$		
*Design A*				
PS	Binomial, logit	$1,X_{1},X_{2},X_{3},X_{4},X_{5}={X_{1}}^{2}$	Binomial, logit	$1,M_{1},M_{2},M_{3},M_{4}$
OR	Poisson, log	$1,X_{1},X_{2},X_{3},X_{4}$	Gaussian, identity	$1,M_{1},M_{2},M_{3},M_{4}$
		$\beta =\left(-0.40,0.60,0.30,0.44,0.30,-0.30\right),\alpha_{0}=\left(9,-0.05,0.05,-0.05\right),\alpha_{1}=\left(10,-0.05,0.05,-0.05\right)$		
		$1,M_{1}=\exp\left(0.10X_{1}\right),M_{2}=X_{2}\left(1+X_{1}\right)+10,M_{3}={\left(0.04X_{3}+0.60\right)}^{2},M_{4}={\left(X_{4}+20\right)}^{2}$		
*Design B*				
PS	Binomial, logit	$1,X_{1},X_{2},X_{3},X_{4},X_{5}={X_{1}}^{2}$	Binomial, logit	$1,M_{1},M_{2},M_{3},M_{4}$
OR	Poisson, log	$1,X_{1},X_{2},X_{3},X_{4}$	Gaussian, identity	$1,M_{1},M_{2},M_{3},M_{4}$
		$\beta =\left(-0.60,0.90,0.45,0.66,0.45,-0.45\right),\alpha_{0}=\left(9,-0.05,0.05,-0.05\right),\alpha_{1}=\left(10,-0.05,0.05,-0.05\right)$		
		$1,M_{1}=\exp\left(0.10X_{1}\right),M_{2}=X_{2}\left(1+X_{1}\right)+10,M_{3}={\left(0.04X_{3}+0.60\right)}^{2},M_{4}={\left(X_{4}+20\right)}^{2}$		
*Design C*				
PS	Binomial, logit	$1,X_{1},X_{2},X_{3},X_{4},X_{5}={X_{1}}^{2}$	Binomial, logit	$1,M_{1},M_{2},M_{3},M_{4}$
OR	Poisson, log	$1,X_{1},X_{2},X_{3},X_{4}$	Gaussian, identity	$1,M_{1},M_{2},M_{3},M_{4}$

#### 5.1.1 Simulation 1

**FIGURE 2:**
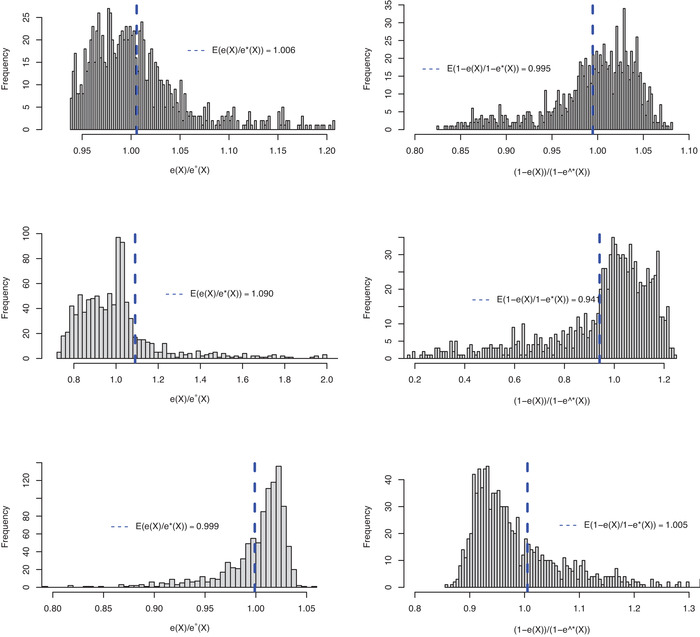
Illustration of the bias reduction in ${\hat{\Delta }}_{{\text{IPW}}_{2}}^{*}$ of the means $E[e\left(X\right)/e^{*}\left(X\right)]$ and $E[\left(1-e\left(X\right)\right)/\left(1-e^{*}\left(X\right)\right)]$ of the PS errors in Designs A, Simulation 1 (top), 2 (middle), and 3 (bottom)

The covariates $\left(X_{1},X_{2},X_{3}\right)$ are generated $X_{1}∼$ Uniform(1,4), $X_{2}∼$ Poisson(3) and $X_{3}∼$ Bernoulli(0.4). Generalized linear models are used to generate a binary treatment *T* and potential outcomes Y(t), t=0,1 with second‐order terms of *X*_1_ and *X*_2_ in both the PS and OR models, see Table 1. The PS distributions for the treated and controls are bounded away from 0 and 1 under the true models and under the model misspecifications (see Figure 2). The PS and OR models (for the AIPW estimator) are stepwise misspecified in the three designs (A, B, C).

**A**: a quadratic term *X*_1_^2^ is omitted in the PS and OR models;
**B**: two quadratic terms, *X*_1_^2^ and *X*_2_^2^, are omitted in the PS and OR models;
**C**: two quadratic terms are omitted and both the OR and PS link functions are misspecified.

#### 5.1.2 Simulation 2

The design is inspired by the simulation study of Funk et al. (2011). We generate the same covariates $\left(X_{1},X_{2},X_{3},X_{4}\right)$ where $X_{1}∼$ Normal(0,1), $X_{2}∼$ Normal(0,1), $X_{3}∼$ Uniform(0.1), and $X_{4}∼$ Normal(0,1). The treatment and outcomes are generated with second‐order terms of *X*_1_ and *X*_2_ in both the PS and OR models given in Table 1. In Figure 4, we see that the PS distributions, under both the true and misspecified models, have poorer overlap and values that are close to 0 and 1. The PS and OR models (for the AIPW estimator) are stepwise misspecified in three designs where:

**A**: two quadratic terms, *X*_1_^2^ and *X*_2_^2^, are omitted in the PS model and *X*_1_^2^ and *X*_3_^2^ in the OR models;
  **FIGURE 4:**
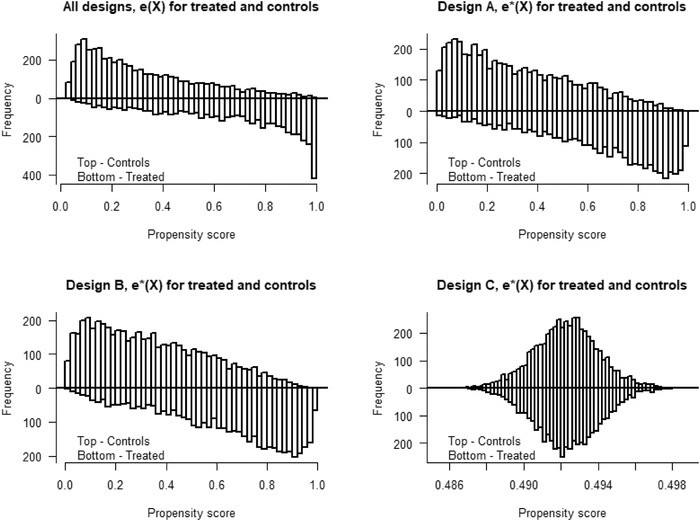
Overlap plots for the propensity score distributions, $\hat{e}\left(X\right)$ and ${\hat{e}}^{*}\left(X\right)$ for treated and controls for Design A (top), B (middle), and C (bottom) in Simulation 2
**B**: two quadratic terms, *X*_1_^2^ and *X*_2_^2^, are omitted in the PS model and *X*_1_^2^ and *X*_3_^2^ in the OR models; and transformations of the first‐order terms, $Z_{1}=X_{1}+X_{2}+X_{3}$ and $Z_{2}=X_{1}+X_{3}+X_{4}$, are applied in the PS and the OR models, respectively;
**C**: two quadratic terms, *X*_1_^2^ and *X*_2_^2^, are omitted in the PS model and *X*_1_^2^ and *X*_3_^2^ in the OR models, *X*_3_ and *X*_4_ are omitted in the PS and the OR models, respectively; and transformations of the first‐order terms, $Z_{3}=X_{1}+X_{2}$ and $Z_{4}=X_{1}+X_{3}$, are applied in the PS and the OR models, respectively.

#### 5.1.3 Simulation 3

**FIGURE 5:**
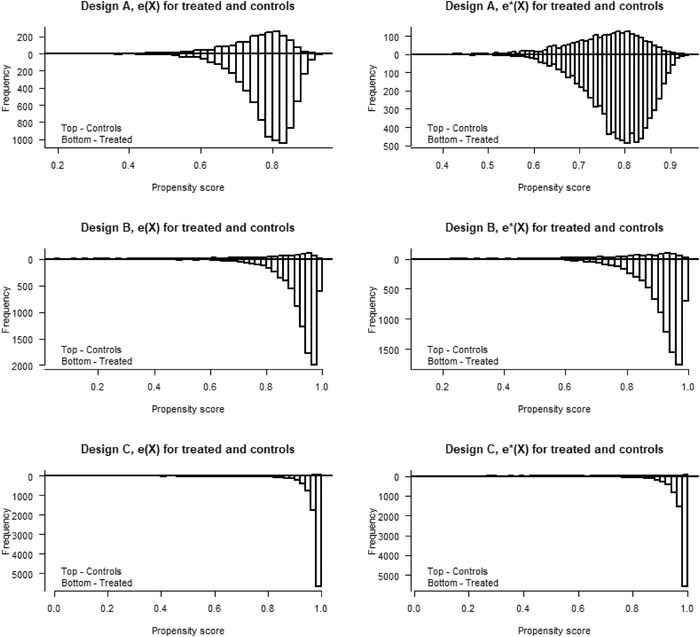
Overlap plots for the propensity score distributions, $\hat{e}\left(X\right)$ and ${\hat{e}}^{*}\left(X\right)$ for treated and controls for Design A (good overlap), B (moderate overlap), and C (poor overlap) in Simulation 3

The design replicates the covariates and PS models of Zhou et al. (2020), in the setting referred to as medium treatment prevalence and PS distributions with good, moderate, and poor overlap (see Figure 5). In our design, the PS and OR models (for the AIPW estimator) are misspecified using the following variable transformations: $M_{2}=X_{2}\left(1+X_{1}\right)+10$, $M_{3}={\left(0.04X_{3}+0.06\right)}^{2}$, and $\left(M_{1},M_{4}\right)=\left(\exp\left(0.10X_{1}\right),{\left(X4+20\right)}^{2}\right)$. Similarly, we generate $X=(X_{1},\text{…},X_{5})$ such that $\left(\begin{array}{c} X_{1}\\ X_{2} \end{array}\right)$ ∼ $\mathrm{Normal}\left[\begin{array}{c} \left(\begin{array}{c} 2\\ 4 \end{array}\right),\left(\begin{array}{cc} 1 & 0.2\\ 0.2 & 1 \end{array}\right) \end{array}\right],\left(\begin{array}{c} X_{3}\\ X_{4} \end{array}\right)∼\mathrm{Normal}\left[\begin{array}{c} \left(\begin{array}{c} 2\\ 4 \end{array}\right),\left(\begin{array}{cc} 1 & 0.2\\ 0.2 & 1 \end{array}\right) \end{array}\right]$. $X_{5}=X_{1}^{2}$

The three simulation designs are:

**A**: good PS distribution overlap, variable transformation, and misspecified link function for the OR model;
**B**: moderate PS distribution overlap, variable transformation, and misspecified link function for the OR model;
**C**: poor PS distribution overlap, variable transformation, and misspecified link function for the OR model.

### 5.2 Results

**FIGURE 3:**
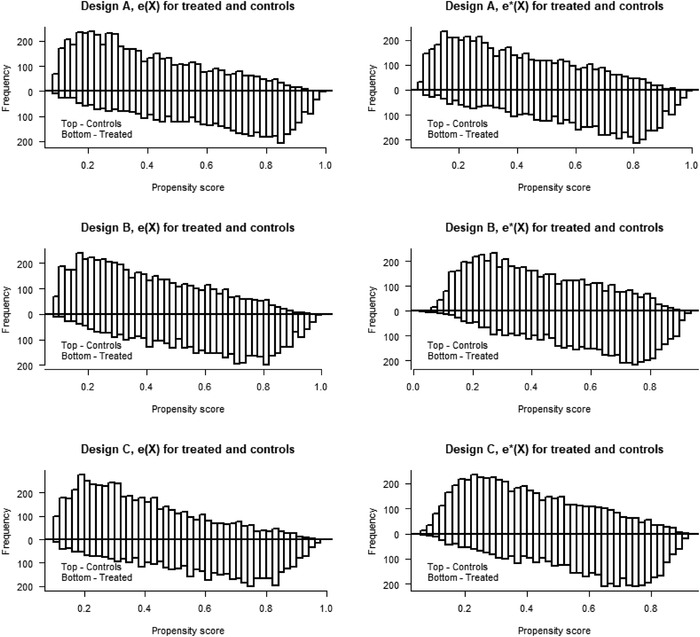
Overlap plots for the propensity score distributions, $\hat{e}\left(X\right)$ and ${\hat{e}}^{*}\left(X\right)$ for treated and controls for Design A (top), B (middle), and C (bottom) in Simulation 1

In Tables 3 and 4, we give the simulation bias, standard error, and mean squared error (MSE) of the three estimators. Tables 5, 6, 7 give numerical approximations for Bias$\left({\hat{\Delta }}_{{\text{IPW}}_{1}}^{*}\right)$, Bias$\left({\hat{\Delta }}_{{\text{IPW}}_{2}}^{*}\right)$, and Bias$\left({\hat{\Delta }}_{\text{AIPW}}^{*}\right)$ using a sample size of n=1,000,000. When using the true models, that is, when studying the estimators ${\hat{\Delta }}_{{\text{IPW}}_{1}}$, ${\hat{\Delta }}_{{\text{IPW}}_{2}}$, and ${\hat{\Delta }}_{\text{AIPW}}$, the bias is small and decreases as the sample size increases. In Simulations 1 and 2, the standard errors follow the expected order with the smallest for ${\hat{\Delta }}_{\text{AIPW}}$ followed by ${\hat{\Delta }}_{{\text{IPW}}_{2}}$ and ${\hat{\Delta }}_{{\text{IPW}}_{1}}$ (Lunceford & Davidian, 2004). In Simulation 3, the standard errors of ${\hat{\Delta }}_{\text{AIPW}}$ and ${\hat{\Delta }}_{{\text{IPW}}_{2}}$ have similar magnitude. Figure 3 gives an illustration of the suppressing effect obtained by the normalization in ${\hat{\Delta }}_{{\text{IPW}}_{2}}^{*}$. For Design A in Simulations 1–3, the figure gives histograms for the simulation model errors $e\left(X\right)/e^{*}\left(X\right)$ and $\left(1-e\left(X\right)\right)/\left(1-e^{*}\left(X\right)\right)$ together with vertical lines for the corresponding means.

**TABLE 3:** Results for Simulations 1 and 2 for sample sizes 500, 1000, and 5000. In Simulation 1, Designs A and B share the same true models. In Simulation 2, the true models are the same in Designs A–C. All true models are given in Table 1.

			Estimators								
			*Simulation 1*								
			${\hat{\Delta }}_{{\text{IPW}}_{1}}^{*}$			${\hat{\Delta }}_{{\text{IPW}}_{2}}^{*}$			${\hat{\Delta }}_{\text{AIPW}}^{*}$		
**n**	Models	Design	Bias	SD	MSE	Bias	SD	MSE	Bias	SD	MSE
500	True	A B	0.030	0.396	0.158	0.010	0.143	0.020	0.003	0.110	0.012
	False	A	0.127	0.410	0.184	0.017	0.142	0.020	0.025	0.118	0.014
	False	B	0.316	0.372	0.238	0.044	0.124	0.017	0.026	0.117	0.014
	True	C	0.011	0.422	0.178	0.010	0.153	0.024	0.009	0.111	0.012
	False	C	0.235	0.336	0.168	−0.051	0.140	0.022	0.047	0.118	0.016
1000	True	A B	0.010	0.254	0.065	0.002	0.098	0.010	−0.003	0.075	0.006
	False	A	0.112	0.270	0.085	0.008	0.097	0.009	0.018	0.079	0.007
	False	B	0.283	0.253	0.144	0.033	0.085	0.008	0.019	0.078	0.006
	True	C	−0.003	0.278	0.077	0.005	0.106	0.011	0.003	0.078	0.006
	False	C	0.219	0.223	0.098	−0.055	0.095	0.012	0.040	0.083	0.008
5000	True	A B	0.002	0.110	0.012	0.001	0.044	0.002	−0.000	0.035	0.001
	False	A	0.108	0.120	0.026	0.009	0.044	0.002	0.022	0.037	0.002
	False	B	0.291	0.107	0.096	0.036	0.039	0.003	0.023	0.036	0.002
	True	C	−0.003	0.123	0.015	0.000	0.044	0.002	−0.000	0.033	0.001
	False	C	0.219	0.098	0.057	−0.060	0.041	0.005	0.038	0.036	0.003

			Estimators								
			*Simulation 2*								
			${\hat{\Delta }}_{{\text{IPW}}_{1}}^{*}$			${\hat{\Delta }}_{{\text{IPW}}_{2}}^{*}$			${\hat{\Delta }}_{\text{AIPW}}^{*}$		
**n**	Models	Design	Bias	SD	MSE	Bias	SD	MSE	Bias	SD	MSE
500	True	A B C	0.006	0.853	0.727	0.033	0.393	0.155	0.010	0.213	0.045
	False	A	0.635	0.650	0.826	0.236	0.334	0.167	0.403	0.708	0.664
	False	B	0.440	0.732	0.728	0.084	0.381	0.152	0.217	0.582	0.385
	False	C	0.608	0.607	0.737	0.271	0.361	0.203	0.597	0.708	0.858
1000	True	A B C	0.011	0.662	0.438	0.029	0.292	0.086	0.013	0.154	0.024
	False	A	0.698	0.724	1.012	0.248	0.286	0.143	0.450	0.824	0.880
	False	B	0.455	0.681	0.669	0.084	0.340	0.123	0.230	0.531	0.335
	False	C	0.619	0.413	0.554	0.266	0.257	0.137	0.606	0.461	0.579
5000	True	A B C	0.004	0.307	0.094	0.010	0.175	0.031	0.010	0.094	0.009
	False	A	0.669	0.217	0.494	0.238	0.129	0.073	0.434	0.266	0.259
	False	B	0.430	0.231	0.238	0.078	0.142	0.026	0.217	0.176	0.078
	False	C	0.646	0.327	0.524	0.282	0.183	0.113	0.650	0.422	0.601

**TABLE 4:** Results for Simulation 3 for sample sizes 500, 1000, and 5000. The true and false models for Designs A–C are described in Table 2.

			Estimators								
			*Simulation 3*								
			${\hat{\Delta }}_{{\text{IPW}}_{1}}^{*}$			${\hat{\Delta }}_{{\text{IPW}}_{2}}^{*}$			${\hat{\Delta }}_{\text{AIPW}}^{*}$		
**n**	Models	Design	Bias	SD	MSE	Bias	SD	MSE	Bias	SD	MSE
500	True	A	−0.011	0.350	0.123	−0.003	0.328	0.108	−0.003	0.329	0.108
	False	A	−0.091	0.367	0.143	0.002	0.332	0.110	−0.001	0.329	0.108
	True	B	−0.050	1.133	1.284	−0.000	0.655	0.429	−0.019	0.697	0.486
	False	B	−1.388	2.402	7.691	0.045	0.778	0.607	0.004	0.856	0.731
	True	C	0.059	3.546	12.566	−0.018	1.260	1.587	−0.033	1.430	2.043
	False	C	−10.041	23.663	660.216	0.077	1.579	2.496	0.031	4.416	19.487
1000	True	A	0.010	0.250	0.063	0.012	0.239	0.057	0.012	0.239	0.057
	False	A	−0.078	0.257	0.072	0.015	0.242	0.059	0.010	0.239	0.057
	True	B	−0.016	0.703	0.493	−0.006	0.440	0.194	−0.008	0.445	0.198
	False	B	−1.379	1.473	4.068	0.031	0.542	0.294	−0.001	0.558	0.311
	True	C	−0.139	2.347	5.522	−0.000	0.952	0.906	−0.002	1.028	1.055
	False	C	−12.065	28.245	942.510	0.118	1.364	1.872	−0.159	6.912	47.752
5000	True	A	0.008	0.110	0.012	0.009	0.107	0.012	0.009	0.107	0.012
	False	A	−0.076	0.114	0.019	0.014	0.108	0.012	0.010	0.107	0.012
	True	B	0.001	0.291	0.085	0.005	0.206	0.042	0.005	0.206	0.043
	False	B	−1.356	0.598	2.197	0.042	0.260	0.069	0.006	0.255	0.065
	True	C	−0.033	0.988	0.976	0.008	0.449	0.202	0.007	0.462	0.213
	False	C	−12.955	12.913	334.401	0.166	0.933	0.898	0.010	3.233	10.442

**TABLE 5:** Asymptotic approximations from Designs A–C in Simulation 1

	Design		
Parameter	A	B	C
μ_1_	11.128	11.128	12.130
$\mu_{1}^{*}$	11.094	11.094	12.098
μ_0_	8.630	8.627	9.633
$\mu_{0}^{*}$	8.579	8.575	9.582
Bias$\left({\hat{\Delta }}_{{\text{IPW}}_{1}}^{*}\right)$	0.106	0.298	0.212
Bias$\left({\hat{\Delta }}_{{\text{IPW}}_{2}}^{*}\right)$	0.009	0.037	−0.058
Bias$\left({\hat{\Delta }}_{\text{AIPW}}^{*}\right)$	0.021	0.025	0.037
Bias${}_{1}\left({\hat{\Delta }}_{{\text{IPW}}_{1}}^{*}\right)$	0.029	0.197	0.130
Bias${}_{1}\left({\hat{\Delta }}_{{\text{IPW}}_{2}}^{*}\right)$	−0.028	0.023	−0.089
Bias${}_{1}\left({\hat{\Delta }}_{\text{AIPW}}^{*}\right)$	0.011	0.013	0.029
$E[\frac{e(X)}{e^{*}\left(X\right)}]$	1.005	1.016	1.019
$\text{cov}[\frac{e(X)}{e^{*}\left(X\right)},\mu_{1}\left(X\right)]$	−0.028	0.022	−0.094
$\text{cov}[\frac{e(X)}{e^{*}\left(X\right)},\mu_{1}^{*}\left(X\right)]$	−0.038	0.009	−0.121
$E\left[\frac{e(X)}{e^{*}\left(X\right)}-1\right]\mu_{1}$	0.057	0.175	0.225
$E[\left(\frac{e(X)}{e^{*}\left(X\right)}-1\right)\mu_{1}\left(X\right)]$	0.029	0.197	0.132
$E[\left(\frac{e(X)}{e^{*}\left(X\right)}-1\right)\mu_{1}^{*}\left(X\right)]$	0.018	0.184	0.104
Bias${}_{2}\left({\hat{\Delta }}_{{\text{IPW}}_{1}}^{*}\right)$	0.078	0.101	0.082
Bias${}_{2}\left({\hat{\Delta }}_{{\text{IPW}}_{2}}^{*}\right)$	0.038	0.014	0.031
Bias${}_{2}\left({\hat{\Delta }}_{\text{AIPW}}^{*}\right)$	0.011	0.012	0.008
$E[\frac{1-e(X)}{1-e^{*}\left(X\right)}]$	0.996	0.990	0.994
$\text{cov}[\frac{1-e(X)}{1-e^{*}\left(X\right)},\mu_{0}\left(X\right)]$	−0.035	−0.015	−0.034
$\text{cov}[\frac{1-e(X)}{1-e^{*}\left(X\right)},\mu_{0}^{*}\left(X\right)]$	−0.025	−0.004	−0.024
$E\left[\frac{1-e(X)}{1-e^{*}\left(X\right)}-1\right]\mu_{0}$	−0.037	−0.088	−0.054
$E[\left(\frac{1-e(X)}{1-e^{*}\left(X\right)}-1\right)\mu_{0}\left(X\right)]$	−0.072	−0.103	−0.087
$E[\left(\frac{1-e(X)}{1-e^{*}\left(X\right)}-1\right)\mu_{0}^{*}\left(X\right)]$	−0.062	−0.091	−0.078

**TABLE 6:** Asymptotic approximations from Designs A–C in Simulation 2

	Design		
Parameter	A	B	C
μ_1_	3.529	3.530	3.535
$\mu_{1}^{*}$	3.460	3.953	3.472
μ_0_	1.337	1.331	1.335
$\mu_{0}^{*}$	1.166	1.398	1.171
Bias$\left({\hat{\Delta }}_{{\text{IPW}}_{1}}^{*}\right)$	0.642	0.422	0.622
Bias$\left({\hat{\Delta }}_{{\text{IPW}}_{2}}^{*}\right)$	0.216	0.060	0.264
Bias$\left({\hat{\Delta }}_{\text{AIPW}}^{*}\right)$	0.397	0.203	0.631
Bias${}_{1}\left({\hat{\Delta }}_{{\text{IPW}}_{1}}^{*}\right)$	0.453	0.325	0.451
Bias${}_{1}\left({\hat{\Delta }}_{{\text{IPW}}_{2}}^{*}\right)$	0.091	0.024	0.150
Bias${}_{1}\left({\hat{\Delta }}_{\text{AIPW}}^{*}\right)$	0.302	0.147	0.532
$E[\frac{e(X)}{e^{*}\left(X\right)}]$	1.102	1.081	1.080
$\text{cov}[\frac{e(X)}{e^{*}\left(X\right)},\mu_{1}\left(X\right)]$	0.102	0.033	0.166
$\text{cov}[\frac{e(X)}{e^{*}\left(X\right)},\mu_{1}^{*}\left(X\right)]$	−0.202	−0.151	−0.359
$E\left[\frac{e(X)}{e^{*}\left(X\right)}-1\right]\mu_{1}$	0.359	0.285	0.283
$E[\left(\frac{e(X)}{e^{*}\left(X\right)}-1\right)\mu_{1}\left(X\right)]$	0.462	0.317	0.449
$E[\left(\frac{e(X)}{e^{*}\left(X\right)}-1\right)\mu_{1}^{*}\left(X\right)]$	0.151	0.168	−0.082
Bias${}_{2}\left({\hat{\Delta }}_{{\text{IPW}}_{1}}^{*}\right)$	0.198	0.097	0.171
Bias${}_{2}\left({\hat{\Delta }}_{{\text{IPW}}_{2}}^{*}\right)$	0.133	0.036	0.113
Bias${}_{2}\left({\hat{\Delta }}_{\text{AIPW}}^{*}\right)$	0.103	0.056	0.100
$E[\frac{1-e(X)}{1-e^{*}\left(X\right)}]$	0.947	0.953	0.952
$\text{cov}[\frac{1-e(X)}{1-e^{*}\left(X\right)},\mu_{0}\left(X\right)]$	−0.124	−0.036	−0.116
$\text{cov}[\frac{1-e(X)}{1-e^{*}\left(X\right)},\mu_{0}^{*}\left(X\right)]$	−0.031	0.024	−0.017
$E\left[\frac{1-e(X)}{1-e^{*}\left(X\right)}-1\right]\mu_{0}$	−0.071	−0.062	−0.064
$E[\left(\frac{1-e(X)}{1-e^{*}\left(X\right)}-1\right)\mu_{0}\left(X\right)]$	−0.195	−0.099	−0.180
$E[\left(\frac{1-e(X)}{1-e^{*}\left(X\right)}-1\right)\mu_{0}^{*}\left(X\right)]$	−0.093	−0.042	−0.073

**TABLE 7:** Asymptotic approximations from Designs A–C in Simulation 3

	Design		
Parameter	A	B	C
μ_1_	9.903	9.899	9.899
$\mu_{1}^{*}$	9.902	9.899	9.898
μ_0_	8.897	8.910	8.900
$\mu_{0}^{*}$	8.895	8.909	8.904
Bias$\left({\hat{\Delta }}_{{\text{IPW}}_{1}}^{*}\right)$	−0.087	−1.349	−12.072
Bias$\left({\hat{\Delta }}_{{\text{IPW}}_{2}}^{*}\right)$	0.005	0.046	0.151
Bias$\left({\hat{\Delta }}_{\text{AIPW}}^{*}\right)$	−0.000	0.009	0.035
Bias${}_{1}\left({\hat{\Delta }}_{{\text{IPW}}_{1}}^{*}\right)$	−0.009	−0.022	−0.006
Bias${}_{1}\left({\hat{\Delta }}_{{\text{IPW}}_{2}}^{*}\right)$	−0.001	−0.001	0.003
Bias${}_{1}\left({\hat{\Delta }}_{\text{AIPW}}^{*}\right)$	−0.000	−0.000	0.003
$E[\frac{e(X)}{e^{*}\left(X\right)}]$	0.999	0.998	0.997
$\text{cov}[\frac{e(X)}{e^{*}\left(X\right)},\mu_{1}\left(X\right)]$	−0.000	−0.001	−0.001
$\text{cov}[\frac{e(X)}{e^{*}\left(X\right)},\mu_{1}^{*}\left(X\right)]$	−0.000	−0.001	−0.001
$E\left[\frac{e(X)}{e^{*}\left(X\right)}-1\right]\mu_{1}$	−0.008	−0.021	−0.030
$E[\left(\frac{e(X)}{e^{*}\left(X\right)}-1\right)\mu_{1}\left(X\right)]$	−0.009	−0.022	−0.031
$E[\left(\frac{e(X)}{e^{*}\left(X\right)}-1\right)\mu_{1}^{*}\left(X\right)]$	−0.009	−0.022	−0.031
Bias${}_{2}\left({\hat{\Delta }}_{{\text{IPW}}_{1}}^{*}\right)$	−0.077	−1.328	−12.040
Bias${}_{2}\left({\hat{\Delta }}_{{\text{IPW}}_{2}}^{*}\right)$	0.006	0.047	0.153
Bias${}_{2}\left({\hat{\Delta }}_{\text{AIPW}}^{*}\right)$	0.000	0.009	0.036
$E[\frac{1-e(X)}{1-e^{*}\left(X\right)}]$	1.009	1.160	2.427
$\text{cov}[\frac{1-e(X)}{1-e^{*}\left(X\right)},\mu_{0}\left(X\right)]$	−0.005	−0.050	−0.376
$\text{cov}[\frac{1-e(X)}{1-e^{*}\left(X\right)},\mu_{0}^{*}\left(X\right)]$	−0.005	−0.046	−0.344
$E\left[\frac{1-e(X)}{1-e^{*}\left(X\right)}-1\right]\mu_{0}$	0.083	1.426	12.714
$E[\left(\frac{1-e(X)}{1-e^{*}\left(X\right)}-1\right)\mu_{0}\left(X\right)]$	0.077	1.376	12.338
$E[\left(\frac{1-e(X)}{1-e^{*}\left(X\right)}-1\right)\mu_{0}^{*}\left(X\right)]$	0.077	1.379	12.366

Under misspecification, the bias in all three simulations and all designs are close to the asymptotic approximations, at least for the largest sample size. For example, in Design C in Simulation 3 with poor overlap, the bias of ${\hat{\Delta }}_{{\text{IPW}}_{2}}^{*}$ is smaller than ${\hat{\Delta }}_{\text{AIPW}}^{*}$ when N=1000 although for N=5000, the bias of ${\hat{\Delta }}_{\text{AIPW}}^{*}$ is smaller than ${\hat{\Delta }}_{{\text{IPW}}_{2}}^{*}$ that is what we see in the asymptotic approximation. For the simulations with poor overlap (both in Simulation 2 and Simulation 3, Design C), the bias is very large because the model misspecification for ${\hat{\Delta }}_{{\text{IPW}}_{1}}$, and both ${\hat{\Delta }}_{{\text{IPW}}_{2}}$ and ${\hat{\Delta }}_{\text{AIPW}}^{*}$ have substantially smaller biases.

In Simulation 1, $\text{Bias}({\hat{\Delta }}_{{\text{IPW}}_{1}}^{*})$ is the largest. $\text{Bias}({\hat{\Delta }}_{{\text{IPW}}_{2}}^{*})$ and $\text{Bias}({\hat{\Delta }}_{\text{AIPW}}^{*})$ are similar, although $\text{Bias}({\hat{\Delta }}_{\text{AIPW}}^{*})$ is slightly smaller in most cases. In Simulation 2, with poorer overlap, $\text{Bias}({\hat{\Delta }}_{{\text{IPW}}_{2}}^{*})$ is the smallest for all three study designs, demonstrating the stabilizing effect of the normalization. Here, in Designs A and B, $\text{Bias}({\hat{\Delta }}_{\text{AIPW}}^{*})$ is smaller than $\text{Bias}({\hat{\Delta }}_{{\text{IPW}}_{1}}^{*})$ but for Design C, they are similar. In Simulation 3, for the largest sample size, $\text{Bias}({\hat{\Delta }}_{\text{AIPW}}^{*})$ is the smallest for all three Designs A–C.

For the MSE in Simulation 1, ${\hat{\Delta }}_{\text{AIPW}}^{*}$ is the smallest; however, the difference with regard to ${\hat{\Delta }}_{{\text{IPW}}_{2}}^{*}$ is not large. In Simulation 2, Designs A and B, the MSE of ${\hat{\Delta }}_{{\text{IPW}}_{2}}^{*}$ is the smallest, followed by the MSE of ${\hat{\Delta }}_{\text{AIPW}}^{*}$ and ${\hat{\Delta }}_{{\text{IPW}}_{1}}^{*}$. For Design C, the MSE of ${\hat{\Delta }}_{\text{AIPW}}^{*}$ is greater than the MSE of ${\hat{\Delta }}_{{\text{IPW}}_{1}}^{*}$. In Simulation 3, the MSE of ${\hat{\Delta }}_{{\text{IPW}}_{2}}^{*}$ and ${\hat{\Delta }}_{\text{AIPW}}^{*}$ is similar for Designs A and B. For Design C, with poor overlap, the MSE is much smaller for ${\hat{\Delta }}_{{\text{IPW}}_{2}}^{*}$.

Studying the two different parts of the biases illustrate how‐not only the variances, but also the biases, get inflated from the lack of overlap in the PS distribution. In Simulation 3 in the design with poor overlap (see Table 7), we have that ${\text{Bias}}_{1}\left({\hat{\Delta }}_{{\text{IPW}}_{1}}^{*}\right)$ is small but ${\text{Bias}}_{2}\left({\hat{\Delta }}_{{\text{IPW}}_{1}}^{*}\right)$ is very large. This result is owing to the ratio $\left(1-e\left(X\right)\right)/\left(1-e^{*}\left(X\right)\right)$ being instable because of sparse data for values close to 0. Correspondingly, we see that the mean $E\left(1-e\left(X\right)\right)/\left(1-e^{*}\left(X\right)\right)$ is far away from 1. In the previous section, we described the stabilizing properties of the normalized IPW, counteracting the model misspecification error. The standard errors under misspecification follow the same pattern as under the true models, which can be expected under regularity conditions from semiparametric theory, see, for example, Boos and Stefanski (2013, Chapter 7.2). In Simulation 2, the standard errors of ${\hat{\Delta }}_{{\text{IPW}}_{2}}^{*}$ are the smallest followed by ${\hat{\Delta }}_{\text{AIPW}}^{*}$ for Designs A and B and ${\hat{\Delta }}_{{\text{IPW}}_{1}}^{*}$ for Design C. In Simulation 3, the standard errors of ${\hat{\Delta }}_{{\text{IPW}}_{2}}^{*}$ are the smallest followed by ${\hat{\Delta }}_{\text{AIPW}}^{*}$ and ${\hat{\Delta }}_{{\text{IPW}}_{1}}^{*}$ for Designs B and C corresponding to moderate and poor PS distribution overlap.

To relate the simulations to the sufficient and necessary conditions derived in Section 4.2, the related expressions from the asymptotic approximations are shown in Tables 5–7. The expectations and covariances that are used for the necessary and sufficient conditions in Equations (11)–(15) can be applied to draw the corresponding conclusions of the estimators. As an example, we see that the necessary condition for the absolute values of Bias${}_{t}\left({\hat{\Delta }}_{\text{AIPW}}^{*}\right)$ to be smaller than the absolute value of Bias${}_{t}\left({\hat{\Delta }}_{{\text{IPW}}_{1}}^{*}\right)$ holds for both t=0, and t=1 in Simulation 1, but the same condition is not satisfied in Simulation 2.

## 6 DATA EXAMPLE

**FIGURE 6:**
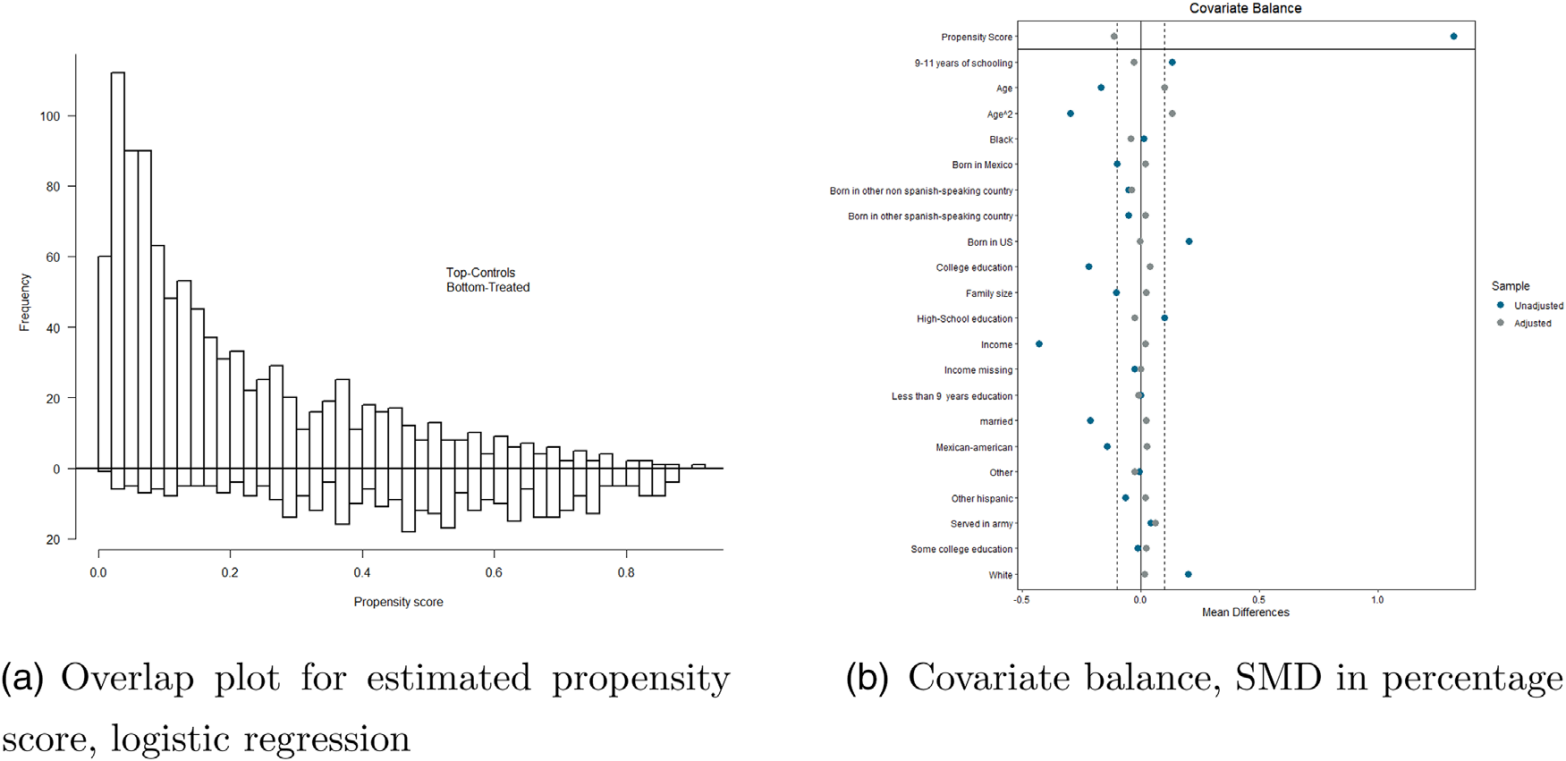
NHANES; data example

As a motivating example, we analyze data from the National Health and Nutrition Examination Survey (NHANES, 2007–2008) for the purpose of estimating the effect of smoking on blood lead levels. Earlier studies have suggested that increased blood lead levels are associated with chronic kidney disease and peripheral arterial diseases (Muntner et al., 2005). Higher blood lead levels are also associated with mortality in the general U.S. population (Menke et al. 2006). The NHANES dataset studied here is a subset of the data previously analyzed by Hsu and Small (2013) evaluating the relationship between smoking and blood lead levels for the treated population (ATT) with a matching approach. To improve overlap for the estimation of the average causal effect, Δ, we select the study population of males, N=1392. The covariate set is also expanded with four more covariates from the original NHANES demographic data. The treated individuals are defined as daily smokers ($N_{1}=386$) and the controls are individuals who had smoked fewer than 100 cigarettes during their life and no cigarettes in the last 30 days ($N_{0}=1006$). The outcome of interest is blood lead levels (micrograms per deciliter, μg/dL). We control for the covariates, age, army service, marriage, birth country, education, family size, and income‐to‐poverty level, see Table 8. For this dataset, we apply the three estimators using a logistic propensity score model and for the AIPW estimator, we additionally use a linear OR model with the same covariates. The overlap is displayed in the mirror histogram of Figure 6a together with balance diagnostics in Figure 6b. Here, we see that the balance reduction achieved from the weighing seems satisfactory for most of the covariates, having standardized mean differences within a balance threshold of 0.10. However, for the age squared, army service, college education group, and the group born in Spanish‐speaking countries other than Mexico, they have standardized mean differences just exceeding this threshold. Applying the simple IPW estimator, ${\hat{\Delta }}_{{\text{IPW}}_{1}}^{*}$, smoking increases the blood lead levels with 1.10 μg/dL (95% CI: 0.63–1.56), whereas the normalized version, ${\hat{\Delta }}_{{\text{IPW}}_{2}}^{*}$ results in a smaller estimated effect of 0.88 μg/dL and a smaller standard error (95% CI: 0.59–1.18). The AIPW estimator, ${\hat{\Delta }}_{\text{AIPW}}^{*}$, further reduces the effect estimate to 0.86 μg/dL and the standard error (95% CI:0.57–1.14, see Table 9). Although applying the estimators to the data does not give information on possible bias due to model misspecification, our results from Section 4 provide guidance to rely on the estimates from ${\hat{\Delta }}_{{\text{IPW}}_{2}}^{*}$ or ${\hat{\Delta }}_{\text{AIPW}}^{*}$, that is, 0.88 or 0.86 μg/dL with corresponding confidence intervals, rather than the higher value from ${\hat{\Delta }}_{{\text{IPW}}_{1}}^{*}$, of 1.10 μg/dL.

**TABLE 8:** Summary statistics for covariates in the NHANES data. Means and sd for lead, age, income‐to‐poverty level and family size, proportions for education, missing income, race, army service, marriage indicator, and birth country

	Nonsmokers	Smokers	Overall
Variables	$N_{0}=1006$	$N_{1}=386$	N=1392
*Blood lead level (*μ*g/dL)*	1.91 (1.70)	2.77 (2.37)	2.15 (1.95)
*Age*	48.7 (17.5)	46.0 (14.7)	48.0 (16.0)
*Education*			
Less than 9th grade	131 (13.0%)	51 (13.2%)	182 (13.1%)
9–11th grade	126 (12.5%)	99 (25.6%)	225 (16.2%)
High school graduate	243 (24.2%)	132 (34.2%)	375 (26.9%)
Some college	238 (23.7%)	86 (22.3%)	324 (23.3%)
College	268 (26.6%)	18 (14.7%)	286 (20.5%)
*Income*			
Income‐to‐poverty level	2.79 (1.55)	2.14 (1.48)	2.61 (1.56)
Missing	86 (8.5%)	23 (6.0%)	109 (7.8%)
Not missing	920 (91.5%)	363 (94.0%)	1283 (92.2%)
*Race*			
White	428 (42.5%)	241 (62.4%)	669 (48.1%)
Black	190(18.9%)	78 (20.2%)	268 (19.3%)
Mexican American	206 (20.5%)	24 (6.2%)	230 (16.5%)
Other Hispanic	120 (11.9%)	22 (5.7%)	142 (10.2%)
Other races	62 (6.2%)	21 (5.4%)	83 (6.0%)
*Served in Army*			
Yes	192 (19.1%)	90 (23.3%)	282 (20.2%)
No	814 (80.9%)	296 (76.7%)	1100 (79.7%)
*Married*			
Yes	640 (46.0%)	164 (11.8%)	804 (57.8%)
No	366 (26.3%)	222 (15.9%)	588 (42.2%)
*Birth country*			
Born in 50 U.S. states or Washington, DC	685(68.0%)	341 (88.3%)	1026 (73.7%)
Born in Mexico	126 (12.5%)	10(2.6%)	136(9.8%)
Born in other Spanish speaking country	97 (9.6%)	17 (4.4%)	116 (8.3%)
Born in other Non‐Spanish speaking country	98 (9.7%)	18 (4.7%)	114 (8.2%)
*Family size*	3.04 (1.67)	2.87 (1.76)	2.99 (1.70)

**TABLE 9:** Results from (A)IPW estimators, effect of smoking on blood lead levels

Estimator	$\hat{\Delta }$	s.e.	95% CI
${\hat{\Delta }}_{{\text{IPW}}_{1}}^{*}$	1.10	0.24	(0.63 − 1.56)
${\hat{\Delta }}_{{\text{IPW}}_{2}}^{*}$	0.88	0.15	(0.59 − 1.18)
${\hat{\Delta }}_{\text{AIPW}}^{*}$	0.86	0.14	(0.57 − 1.14)

## 7 DISCUSSION

In this paper, we investigate biases of two IPW estimators and an AIPW estimator under model misspecification. For this purpose, we use a generic probability limit, under misspecification of the PS and OR models, which exists under general conditions. Since the PS enters the estimator in different ways for the IPW estimators under study, the consequences of the model misspecification are not the same. The bias of the IPW estimators depends on the covariance between the PS‐model error and the conditional outcome in different ways and the resulting bias can be in opposite directions. For the IPW estimators, normalization has the potential of reducing the bias because it scales the estimator in a mitigating manner. Comparing the bias of the AIPW estimator with a simple IPW estimator, the necessary condition for the AIPW estimator to have a smaller bias is that the expectation of the outcome model under misspecification is less than twice the true conditional outcome, where the expectations include a scaling with the PS‐model error. For comparison with the normalized IPW estimator, the (PS‐error scaled) misspecified outcome involves an interval defined by the true conditional outcome adding and subtracting the absolute value of the covariance between the PS‐model error and the conditional outcome.

The biases and conditions are exemplified in three simulation studies where the fitted misspecified models fails in specifying nonlinearities, functional form (through misspecified link functions), and covariates. The simulation studies are also accompanied by numerical approximations of the large‐sample biases. The third simulation study specifically compares the impact of good, moderate, and poor overlap on the bias due to model misspecification. Here, we see that it is not only the variance that gets inflated from PS values close to 0 or 1, but the bias due to model misspecification also increases rapidly. The normalized IPW and AIPW estimators show a more stable performance. The bias expressions of the IPW and AIPW estimators suggest that the AIPW estimator has a smaller bias than the IPW estimators even under moderate misspecification of the outcome model. For the AIPW estimator, poor overlap and large differences between e(X) and $e^{*}\left(X\right)$ are compensated for by outcome model assumptions in the area where data are sparse. However, in the simulations, the normalized IPW estimator also performs well due to the implicit stabilization from the PS‐model errors. Since all biases include the PS‐model error, we suggest that a researcher should be careful when modeling the PS even though an OR model is additionally involved.

### OPEN RESEARCH BADGES

This article has earned an Open Data badge for making publicly available the digitally‐shareable data necessary to reproduce the reported results. The data is available in the Supporting Information section.

This article has earned an open data badge “**Reproducible Research**” for making publicly available the code necessary to reproduce the reported results. The results reported in this article could fully be reproduced.

## Supporting information

Supporting Information.

## Data Availability

The data that support the findings of this study are openly available at https://github.com/IngWae/Bias_AIPW

## APPENDIX A

### A.1 Regularity conditions

The convergence in probability of ${\hat{\Delta }}_{{\text{IPW}}_{1}}^{*}$, ${\hat{\Delta }}_{{\text{IPW}}_{2}}^{*}$, and ${\hat{\Delta }}_{\text{AIPW}}^{*}$ to their corresponding expectations would follow directly from a weak law of large numbers (WLLN) for an iid sample of $\left(T_{i},X_{i},Y_{i}\right)$ except for the estimated parameters ${\hat{\beta }}^{*}$ in ${\hat{e}}^{*}\left(X_{i}\right)$ and ${\hat{\alpha }}_{t}^{*}$ in ${\hat{\mu }}_{t}^{*}\left(X_{i}\right)$, t=0,1. To justify the biases in Section 4, consider a general representation of a function $g[T,Y,X,\hat{\theta }]$ where $\hat{\theta }\overset{p}{⟶}\theta_{0}$ and

(A1)
$$\frac{1}{n}\sum g\left[T_{i},X_{i},Y_{i},\hat{\theta }\right]\overset{p}{⟶}E\left[g(T,X,Y,\theta_{0})\right].$$

The $\hat{\theta }$ in (A1) corresponds to ${\hat{\beta }}^{*}$ for ${\hat{\Delta }}_{{\text{IPW}}_{1}}^{*}$, ${\hat{\Delta }}_{{\text{IPW}}_{2}}^{*}$, and $\left({\hat{\alpha }}^{*},{\hat{\beta }}^{*}\right)$ for ${\hat{\Delta }}_{\text{AIPW}}^{*}$ and under Assumptions 5 and 7, the consistency of $\hat{\theta }$ is ensured. Regularity conditions for the function *g* can be given, see, for example, Boos and Stefanski (2013, Theorem 7.3) who show that (A1) holds for differentiable functions with bounded derivatives (w.r.t. θ). The regularity conditions for $g[X_{i},T_{i},Y_{i},\hat{\theta }]$, for the three estimators, imply conditions on the models $e^{*}\left(X,\beta^{*}\right)$ and $\mu_{t}^{*}\left(X,\alpha_{t}^{*}\right)$ such that the regularity condition for *g* is satisfied. Under (A1), we can insert the limiting values $\beta^{*}$ and $\alpha^{*}$ and their corresponding $e^{*}\left(X\right)$ and $\mu_{t}^{*}\left(X\right)$, t=0,1 when taking a WLLN.

### A.2 Biases

Under the regularity conditions and Assumptions 5–6 (IPW) and 5–7 (AIPW), we derive the bias in Equation (4). For ${\hat{\Delta }}_{{\text{IPW}}_{1}}^{*}$:

$$\frac{1}{n}\sum_{i=1}^{n}\frac{T_{i}Y_{i}}{{\hat{e}}^{*}\left(X_{i}\right)}-\frac{1}{n}\sum_{i=1}^{n}\frac{\left(1-T_{i}\right)Y_{i}}{1-{\hat{e}}^{*}\left(X_{i}\right)}\overset{p}{⟶}E\left[\frac{TY}{e^{*}\left(X\right)}\right]-E\left[\frac{\left(1-T\right)Y}{\left(1-e^{*}\left(X\right)\right)}\right],$$

and

$$\begin{array}{cc} E\left[\frac{TY}{e^{*}\left(X\right)}\right]-E\left[\frac{\left(1-T\right)Y}{\left(1-e^{*}\left(X\right)\right)}\right] & =E\left[E\left[\frac{TY}{e^{*}\left(X\right)}|X\right]\right]-E\left[E\left[\frac{\left(1-T\right)Y}{\left[1-e^{*}\left(X\right)\right]}|X\right]\right]\\ & =E\left[\frac{e(X)}{e^{*}\left(X\right)}\mu_{1}\left(X\right)\right]-E\left[\frac{\left(1-e(X)\right)}{\left(1-e^{*}\left(X\right)\right)}\mu_{0}\left(X\right)\right], \end{array}$$

subtracting with Δ gives

$$\begin{array}{cc} \text{Bias}({\hat{\Delta }}_{{\text{IPW}}_{1}}^{*})= & E\left[\frac{e(X)}{e^{*}\left(X\right)}\mu_{1}\left(X\right)\right]-E\left[\frac{1-e(X)}{1-e^{*}\left(X\right)}\mu_{0}\left(X\right)\right]-\left(\mu_{1}-\mu_{0}\right). \end{array}$$

The biases of Equations (5) and (6) are derived similarly.

### A.3 Comparisons

To study the consequences of model misspecification for the estimators, we compare each difference involving $\mu_{1}\left(X\right)$ and $\mu_{0}\left(X\right)$ separately. For example, we study the bias of ${\hat{\Delta }}_{{\text{IPW}}_{1}}^{*}$

$$\begin{array}{cc} {\hat{\Delta }}_{{\text{IPW}}_{1}}^{*}-\Delta \overset{p}{⟶} & E\left[\frac{e(X)}{e^{*}\left(X\right)}\mu_{1}\left(X\right)\right]-E\left[\frac{1-e(X)}{1-e^{*}\left(X\right)}\mu_{0}\left(X\right)\right]-\left(\mu_{1}-\mu_{0}\right)\\ = & E\left[\frac{e(X)}{e^{*}\left(X\right)}\mu_{1}\left(X\right)\right]-\mu_{1}+\mu_{0}-E\left[\frac{1-e(X)}{1-e^{*}\left(X\right)}\mu_{0}\left(X\right)\right]. \end{array}$$

Since the errors $e\left(X\right)/e^{*}\left(X\right)$ and $\left(1-e\left(X\right)\right)/\left(1-e^{*}\left(X\right)\right)$ are inversely related, we would normally not expect that the biases from the two parts $E\left[\frac{e(X)}{e^{*}\left(X\right)}\mu_{1}\left(X\right)\right]-\mu_{1}$ and $\mu_{0}-E\left[\frac{1-e(X)}{1-e^{*}\left(X\right)}\mu_{0}\left(X\right)\right]$ cancel each other out. For example, if $E\left[\frac{e(X)}{e^{*}\left(X\right)}\mu_{1}\left(X\right)\right]-\mu_{1}>0$, then we might expect that $\mu_{0}-E\left[\frac{1-e(X)}{1-e^{*}\left(X\right)}\mu_{0}\left(X\right)\right]>0$ and similarly for negative values. However, if there is large effect modification on the difference scale, the two components might be in different directions.

Inequalities concerning the biases are made with respect to the absolute values for two parts separately, for example, for Bias$\left({\hat{\Delta }}_{{\text{IPW}}_{1}}^{*}\right)$, we investigate

(A2)
$$\left|{\text{Bias}}_{1}\left({\hat{\Delta }}_{{\text{IPW}}_{1}}^{*}\right)\right|=\left|E\left[\frac{e(X)}{e^{*}\left(X\right)}\mu_{1}\left(X\right)\right]-\mu_{1}\right|.$$

Since |−a|=|a|, the second part is

(A3)
$$\left|{\text{Bias}}_{2}\left({\hat{\Delta }}_{{\text{IPW}}_{1}}^{*}\right)\right|=\left|E\left[\frac{1-e(X)}{1-e^{*}\left(X\right)}\mu_{0}\left(X\right)\right]-\mu_{0}\right|,$$

and similarly for (8). The conditions derived for the first part of the biases, (7) and (8), can be directly applied to the second part of the biases replacing $e\left(X\right)/e^{*}\left(X\right)$, with $\left(1-e\left(X\right)\right)/\left(1-e^{*}\left(X\right)\right)$ and $\mu_{1}\left(X\right)$ with $\mu_{0}\left(X\right)$. In a similar manner, we have for Bias${}_{2}\left({\hat{\Delta }}_{\text{AIPW}}^{*}\right)$

$$\left|E\left[\frac{\left(1-e\left(X\right)\right)-\left(1-e^{*}\left(X\right)\right)\left(\mu_{0}\left(X\right)-\mu_{0}^{*}\left(X\right)\right)}{\left(1-e^{*}\left(X\right)\right)}\right]\right|=\left|E\left[\frac{\left(e\left(X\right)-e^{*}\left(X\right)\right)\left(\mu_{0}\left(X\right)-\mu_{0}^{*}\left(X\right)\right)}{\left(1-e^{*}\left(X\right)\right)}\right]\right|,$$

and the conditions derived for the first part of the bias, (9), can be directly applied to (A3) additionally replacing $\mu_{1}^{*}\left(X\right)$ with $\mu_{0}^{*}\left(X\right)$.

*Necessary and sufficient condition for* $|{\mathrm{Bias}}_{1}\left({\hat{\Delta }}_{{\text{IPW}}_{2}}^{*}\right)\left|<\right|{\mathrm{Bias}}_{1}\left({\hat{\Delta }}_{{\text{IPW}}_{1}}^{*}\right)|$ *in* (10)

$$\left|\frac{E\left[\frac{e(X)}{e^{*}\left(X\right)}\mu_{1}\left(X\right)\right]}{E\left[\frac{e(X)}{e^{*}\left(X\right)}\right]}-\mu_{1}\right|<E\left[\frac{e(X)}{e^{*}\left(X\right)}\mu_{1}\left(X\right)\right]-\mu_{1}.$$

1. Assuming that $E[\frac{e(X)}{e^{*}\left(X\right)}\mu_{1}\left(X\right)-\mu_{1}]>0$:
  We have
  (A4)
  $$-E\left[\frac{e(X)}{e^{*}\left(X\right)}\right]\left(E\left[\frac{e(X)}{e^{*}\left(X\right)}\mu_{1}\left(X\right)\right]-\mu_{1}\right)<\text{cov}\left[\frac{e(X)}{e^{*}\left(X\right)},\mu_{1}\left(X\right)\right]<E\left[\frac{e(X)}{e^{*}\left(X\right)}\right]\left(E\left[\frac{e(X)}{e^{*}\left(X\right)}\mu_{1}\left(X\right)\right]-\mu_{1}\right).$$
2. Assuming that $E\left[\frac{e(X)}{e^{*}\left(X\right)}\mu_{1}\left(X\right)\right]-\mu_{1}<0$:
  $$\left|\frac{E\left[\frac{e(X)}{e^{*}\left(X\right)}\mu_{1}\left(X\right)\right]}{E\left[\frac{e(X)}{e^{*}\left(X\right)}\right]}-\mu_{1}\right|<-E\left[\frac{e(X)}{e^{*}\left(X\right)}\mu_{1}\left(X\right)\right]-\mu_{1}.$$
  Here, we have
  (A5)
  $$E\left[\frac{e(X)}{e^{*}\left(X\right)}\right]\left(E\left[\frac{e(X)}{e^{*}\left(X\right)}\mu_{1}\left(X\right)\right]-\mu_{1}\right)<\text{cov}\left[\frac{e(X)}{e^{*}\left(X\right)},\mu_{1}\left(X\right)\right]<-E\left[\frac{e(X)}{e^{*}\left(X\right)}\right]\left(E\left[\frac{e(X)}{e^{*}\left(X\right)}\mu_{1}\left(X\right)\right]-\mu_{1}\right),$$
  by A4 and A5, we have
  $|\text{cov}\left[\frac{e(X)}{e^{*}\left(X\right)},\mu_{1}\left(X\right)\right]\left|<\right|E\left[\frac{e(X)}{e^{*}\left(X\right)}\right]\left(E\left[\frac{e(X)}{e^{*}\left(X\right)}\mu_{1}\left(X\right)\right]-\mu_{1}|\right)$.

*Necessary condition for* $|{\mathrm{Bias}}_{1}\left({\hat{\Delta }}_{\text{AIPW}}^{*}\right)\left|<\right|{\mathrm{Bias}}_{1}\left({\hat{\Delta }}_{{\text{IPW}}_{1}}^{*}\right)|$ *in* (12)

1. Assuming that $E[\left(\frac{e(X)}{e^{*}\left(X\right)}-1\right)\mu_{1}\left(X\right)]>0$:
  $$\left|E\left[\left(\frac{e(X)}{e^{*}\left(X\right)}-1\right)\left(\mu_{1}\left(X\right)-\mu_{1}^{*}\left(X\right)\right)\right]\right|<E\left[\left(\frac{e(X)}{e^{*}\left(X\right)}-1\right)\mu_{1}\left(X\right)\right].$$
  Here, we have
  $$\begin{array}{cc} -E\left[\left(\frac{e(X)}{e^{*}\left(X\right)}-1\right)\mu_{1}\left(X\right)\right] & <E\left[\left(\frac{e(X)}{e^{*}\left(X\right)}-1\right)\left(\mu_{1}\left(X\right)-\mu_{1}^{*}\left(X\right)\right)\right]<E\left[\left(\frac{e(X)}{e^{*}\left(X\right)}-1\right)\mu_{1}\left(X\right)\right], \end{array}$$
  and
  (A6)
  $$0<E\left[\left(\frac{e(X)}{e^{*}\left(X\right)}-1\right)\mu_{1}^{*}\left(X\right)\right]<2\cdot E\left[\left(\frac{e(X)}{e^{*}\left(X\right)}-1\right)\mu_{1}\left(X\right)\right].$$
2. Assuming that $E[\left(\frac{e(X)}{e^{*}\left(X\right)}-1\right)\mu_{1}\left(X\right)]<0$:
  $$\left|E\left[\left(\frac{e(X)}{e^{*}\left(X\right)}-1\right)\left(\mu_{1}\left(X\right)-\mu_{1}^{*}\left(X\right)\right)\right]\right|<-E\left[\left(\frac{e(X)}{e^{*}\left(X\right)}-1\right)\mu_{1}\left(X\right)\right].$$
  Here, we have
  (A7)
  $$\begin{array}{cc} E\left[\left(\frac{e(X)}{e^{*}\left(X\right)}-1\right)\mu_{1}\left(X\right)\right] & <E\left[\left(\frac{e(X)}{e^{*}\left(X\right)}-1\right)\left(\mu_{1}\left(X\right)-\mu_{1}^{*}\left(X\right)\right)\right]<-E\left[\left(\frac{e(X)}{e^{*}\left(X\right)}-1\right)\mu_{1}\left(X\right)\right],\\ 2\cdot E\left[\left(\frac{e(X)}{e^{*}\left(X\right)}-1\right)\mu_{1}\left(X\right)\right] & <E\left[\left(\frac{e(X)}{e^{*}\left(X\right)}-1\right)\mu_{1}^{*}\left(X\right)\right]<0, \end{array}$$
  and by (A6) and (A7),
  $$\begin{array}{c} \left|E\left[\left(\frac{e(X)}{e^{*}\left(X\right)}-1\right)\mu_{1}^{*}\left(X\right)\right]\right|\leq 2\cdot \left|E\left[\left(\frac{e(X)}{e^{*}\left(X\right)}-1\right)\mu_{1}\left(X\right)\right]\right|. \end{array}$$

*Sufficient condition for* $|{\mathrm{Bias}}_{1}\left({\hat{\Delta }}_{\text{AIPW}}^{*}\right)\left|<\right|{\mathrm{Bias}}_{1}\left({\hat{\Delta }}_{{\text{IPW}}_{1}}^{*}\right)|$ *in* (13)

The sufficient condition follows from the above by adding to the necessary condition that

$$E\left[\left(\frac{e(X)}{e^{*}\left(X\right)}-1\right)\mu_{1}^{*}\left(X\right)\right]\;\text{and}\;E\left[\left(\frac{e(X)}{e^{*}\left(X\right)}-1\right)\mu_{1}\left(X\right)\right]$$

are either both positive or both negative, since then either (A6) or (A7) holds.

*Necessary condition of Equation* 14

1. Assuming that:
  $$\text{cov}\left[\frac{e(X)}{e^{*}\left(X\right)},\mu_{1}\left(X\right)\right]>0\Leftrightarrow \frac{E\left[\frac{e(X)}{e^{*}\left(X\right)}\mu_{1}\left(X\right)\right]}{E\left[\frac{e(X)}{e^{*}\left(X\right)}\right]}-\mu_{1}>0.$$
  Here, we have
  $$\begin{array}{cc} -\frac{E\left[\frac{e(X)}{e^{*}\left(X\right)}\mu_{1}\left(X\right)\right]}{E\left[\frac{e(X)}{e^{*}\left(X\right)}\right]}+\mu_{1} & <E\left[\left(\frac{e(X)}{e^{*}\left(X\right)}-1\right)\left(\mu_{1}\left(X\right)-\mu_{1}^{*}\left(X\right)\right)\right]<\frac{E\left[\frac{e(X)}{e^{*}\left(X\right)}\mu_{1}\left(X\right)\right]}{E\left[\frac{e(X)}{e^{*}\left(X\right)}\right]}-\mu_{1},\\ -\text{cov}\left[\frac{e(X)}{e^{*}\left(X\right)},\mu_{1}\left(X\right)\right] & <E\left[\frac{e(X)}{e^{*}\left(X\right)}\right]E\left[\left(\frac{e(X)}{e^{*}\left(X\right)}-1\right)\left(\mu_{1}\left(X\right)-\mu_{1}^{*}\left(X\right)\right)\right]<\text{cov}\left[\frac{e(X)}{e^{*}\left(X\right)},\mu_{1}\left(X\right)\right], \end{array}$$
  and
  (A8)
  $$\begin{array}{c} \begin{array}{c} E\left[\left(\frac{e(X)}{e^{*}\left(X\right)}-1\right)\mu_{1}\left(X\right)\right]-\frac{\text{cov}\left[\frac{e(X)}{e^{*}\left(X\right)},\mu_{1}\left(X\right)\right]}{E\left[\frac{e(X)}{e^{*}\left(X\right)}\right]}<E\left[\left(\frac{e(X)}{e^{*}\left(X\right)}-1\right)\mu_{1}^{*}\left(X\right)\right]<E\left[\left(\frac{e(X)}{e^{*}\left(X\right)}-1\right)\mu_{1}\left(X\right)\right]+\frac{\text{cov}\left[\frac{e(X)}{e^{*}\left(X\right)},\mu_{1}\left(X\right)\right]}{E\left[\frac{e(X)}{e^{*}\left(X\right)}\right]}. \end{array} \end{array}$$
2. Assuming that:
  $$\text{cov}\left[\frac{e(X)}{e^{*}\left(X\right)},\mu_{1}\left(X\right)\right]<0\Leftrightarrow \frac{E\left[\frac{e(X)}{e^{*}\left(X\right)}\mu_{1}\left(X\right)\right]}{E\left[\frac{e(X)}{e^{*}\left(X\right)}\right]}-\mu_{1}<0,$$
  it follows that
  $$\begin{array}{cc} \frac{E\left[\frac{e(X)}{e^{*}\left(X\right)}\mu_{1}\left(X\right)\right]}{E\left[\frac{e(X)}{e^{*}\left(X\right)}\right]}-\mu_{1} & <E\left[\left(\frac{e(X)}{e^{*}\left(X\right)}-1\right)\left(\mu_{1}\left(X\right)-\mu_{1}^{*}\left(X\right)\right)\right]<-\frac{E\left[\frac{e(X)}{e^{*}\left(X\right)}\mu_{1}\left(X\right)\right]}{E\left[\frac{e(X)}{e^{*}\left(X\right)}\right]}+\mu_{1}\\ \text{cov}\left[\frac{e(X)}{e^{*}\left(X\right)},\mu_{1}\left(X\right)\right] & <E\left[\frac{e(X)}{e^{*}\left(X\right)}\right]E\left[\left(\frac{e(X)}{e^{*}\left(X\right)}-1\right)\left(\mu_{1}\left(X\right)-\mu_{1}^{*}\left(X\right)\right)\right]<-cov\left[\frac{e(X)}{e^{*}\left(X\right)},\mu_{1}\left(X\right)\right], \end{array}$$
  and
  (A9)
  $$\begin{array}{c} \begin{array}{c} E\left[\left(\frac{e(X)}{e^{*}\left(X\right)}-1\right)\mu_{1}\left(X\right)\right]+\frac{\text{cov}\left[\frac{e(X)}{e^{*}\left(X\right)},\mu_{1}\left(X\right)\right]}{E\left[\frac{e(X)}{e^{*}\left(X\right)}\right]}<E\left[\left(\frac{e(X)}{e^{*}\left(X\right)}-1\right)\mu_{1}^{*}\left(X\right)\right]<E\left[\left(\frac{e(X)}{e^{*}\left(X\right)}-1\right)\mu_{1}\left(X\right)\right]-\frac{cov\left[\frac{e(X)}{e^{*}\left(X\right)},\mu_{1}\left(X\right)\right]}{E\left[\frac{e(X)}{e^{*}\left(X\right)}\right]}, \end{array} \end{array}$$
  by (A8) and (A9), we have that
  $$\begin{array}{c} E\left[\left(\frac{e(X)}{e^{*}\left(X\right)}-1\right)\mu_{1}\left(X\right)\right]-\frac{\left|\text{cov}\left[\frac{e(X)}{e^{*}\left(X\right)},\mu_{1}\left(X\right)\right]\right|}{E\left[\frac{e(X)}{e^{*}\left(X\right)}\right]}<E\left[\left(\frac{e(X)}{e^{*}\left(X\right)}-1\right)\mu_{1}^{*}\left(X\right)\right]<E\left[\left(\frac{e(X)}{e^{*}\left(X\right)}-1\right)\mu_{1}\left(X\right)\right]+\frac{\left|\text{cov}\left[\frac{e(X)}{e^{*}\left(X\right)},\mu_{1}\left(X\right)\right]\right|}{E\left[\frac{e(X)}{e^{*}\left(X\right)}\right]}, \end{array}$$

*Sufficient condition of* (15)

If $E[\left(\frac{e(X)}{e^{*}\left(X\right)}-1\right)\mu_{1}\left(X\right)]$ and $\text{cov}[\frac{e(X)}{e^{*}\left(X\right)},\mu_{1}\left(X\right)]$ are both positive

then,

$$\begin{array}{cc} E\left[\left(\frac{e(X)}{e^{*}\left(X\right)}-1\right)\mu_{1}\left(X\right)\right]+\left|\text{cov}\left[\frac{e(X)}{e^{*}\left(X\right)},\mu_{1}\left(X\right)\right]\right| & =\left|E\left[\left(\frac{e(X)}{e^{*}\left(X\right)}-1\right)\mu_{1}\left(X\right)\right]+\text{cov}\left[\frac{e(X)}{e^{*}\left(X\right)},\mu_{1}\left(X\right)\right]\right| \end{array}$$

and

$$\begin{array}{cc} -\left|E\left\{\left[\frac{e(X)}{e^{*}\left(X\right)}-1\right]\mu_{1}\left(X\right)\right\}+\text{cov}\left[\frac{e(X)}{e^{*}\left(X\right)},\mu_{1}\left(X\right)\right]\right| & <E\left[\left(\frac{e(X)}{e^{*}\left(X\right)}-1\right)\mu_{1}\left(X\right)\right]+\left|\text{cov}\left[\frac{e(X)}{e^{*}\left(X\right)},\mu_{1}\left(X\right)\right]\right|. \end{array}$$

If $E[\left(\frac{e(X)}{e^{*}\left(X\right)}-1\right)\mu_{1}\left(X\right)]$ and $\text{cov}[\frac{e(X)}{e^{*}\left(X\right)},\mu_{1}\left(X\right)]$ are both negative, then we have

$$\begin{array}{cc} E\left[\left(\frac{e(X)}{e^{*}\left(X\right)}-1\right)\mu_{1}\left(X\right)\right]+\left|\text{cov}\left[\frac{e(X)}{e^{*}\left(X\right)},\mu_{1}\left(X\right)\right]\right| & <\left|E\left[\left(\frac{e(X)}{e^{*}\left(X\right)}-1\right)\mu_{1}\left(X\right)\right]+\text{cov}\left[\frac{e(X)}{e^{*}\left(X\right)},\mu_{1}\left(X\right)\right]\right|, \end{array}$$

and

$$\begin{array}{cc} -\left|E\left[\left(\frac{e(X)}{e^{*}\left(X\right)}-1\right)\mu_{1}\left(X\right)\right]+\text{cov}\left[\frac{e(X)}{e^{*}\left(X\right)},\mu_{1}\left(X\right)\right]\right| & =E\left[\left(\frac{e(X)}{e^{*}\left(X\right)}-1\right)\mu_{1}\left(X\right)\right]+\left|\text{cov}\left[\frac{e(X)}{e^{*}\left(X\right)},\mu_{1}\left(X\right)\right]\right|, \end{array}$$

and the necessary condition from Equation (14) follows.
