# Supplementary figures and images for: Model misspecification and bias for inverse probability weighting estimators of average causal effects
Source: Biom J. 2022 Aug 31;65(2):2100118. doi: 10.1002/bimj.202100118 (PMC10087564; doi:10.1002/bimj.202100118)

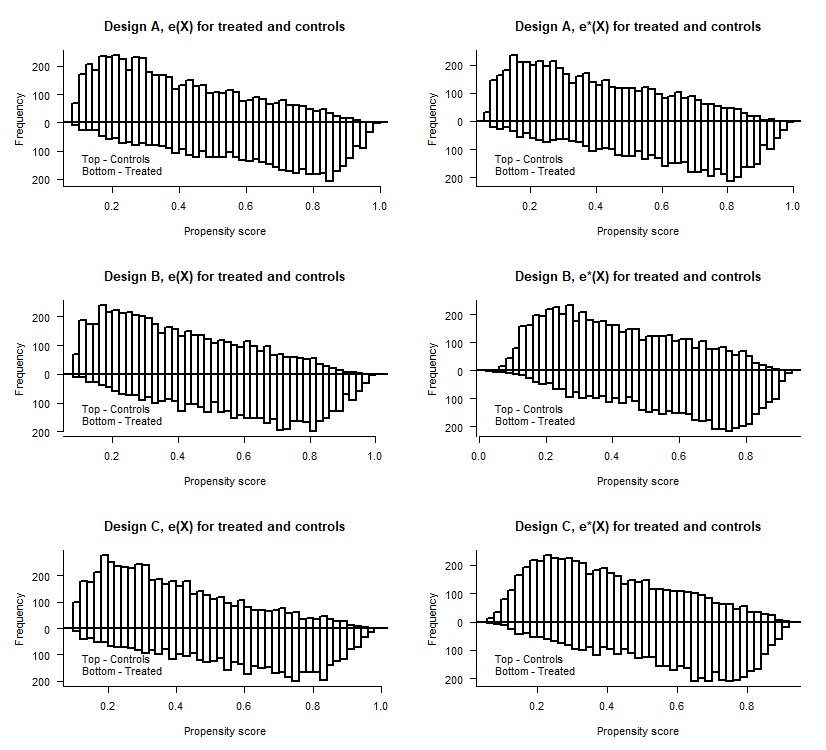

Supplement: Supplementary file 1 — Supporting Information. [file BIMJ-65-0-s001.zip › Code_and_Data/results/Figure_3.png]

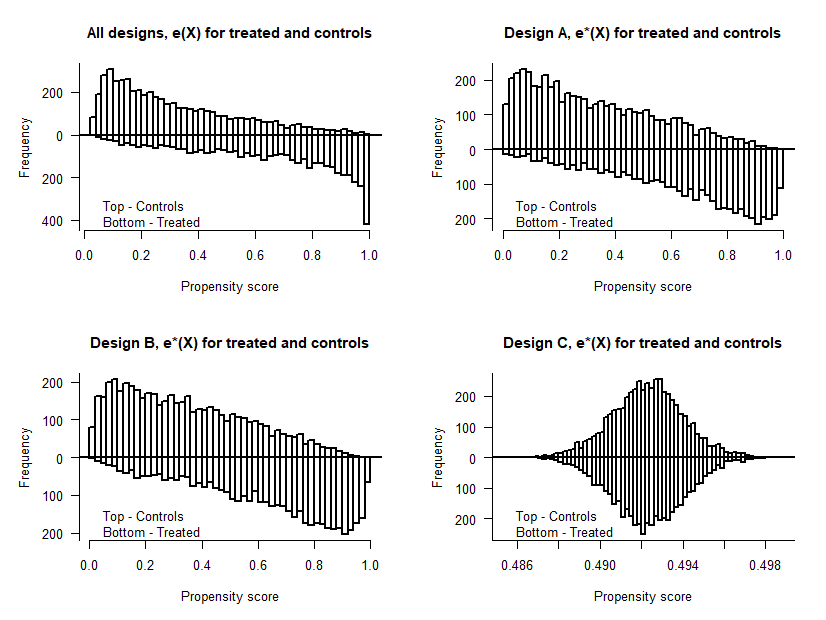

Supplement: Supplementary file 1 — Supporting Information. [file BIMJ-65-0-s001.zip › Code_and_Data/results/Figure_4.png]

PS-models

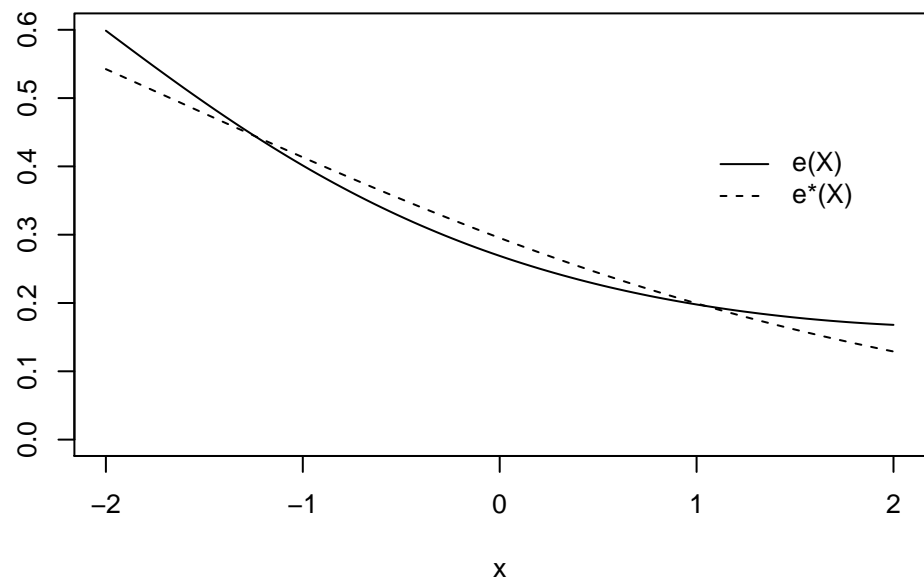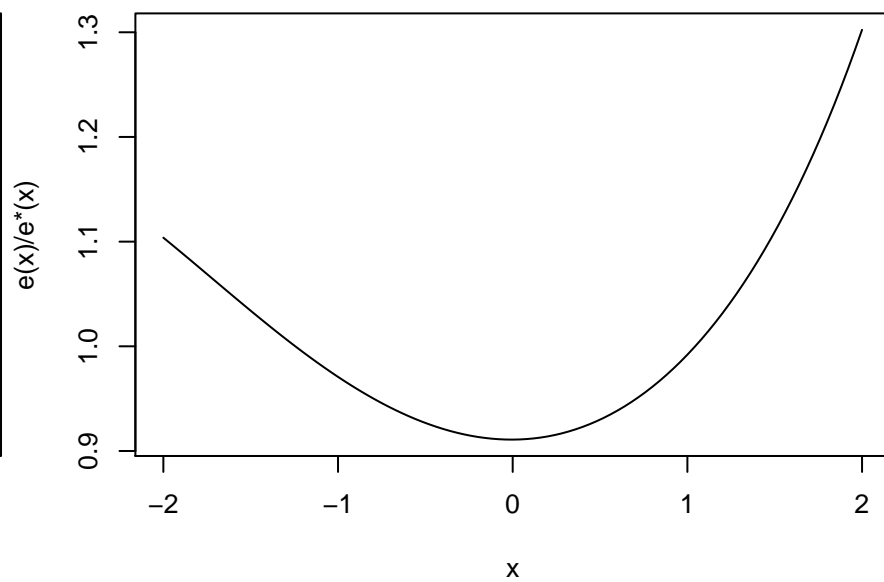

Conditional outcomes

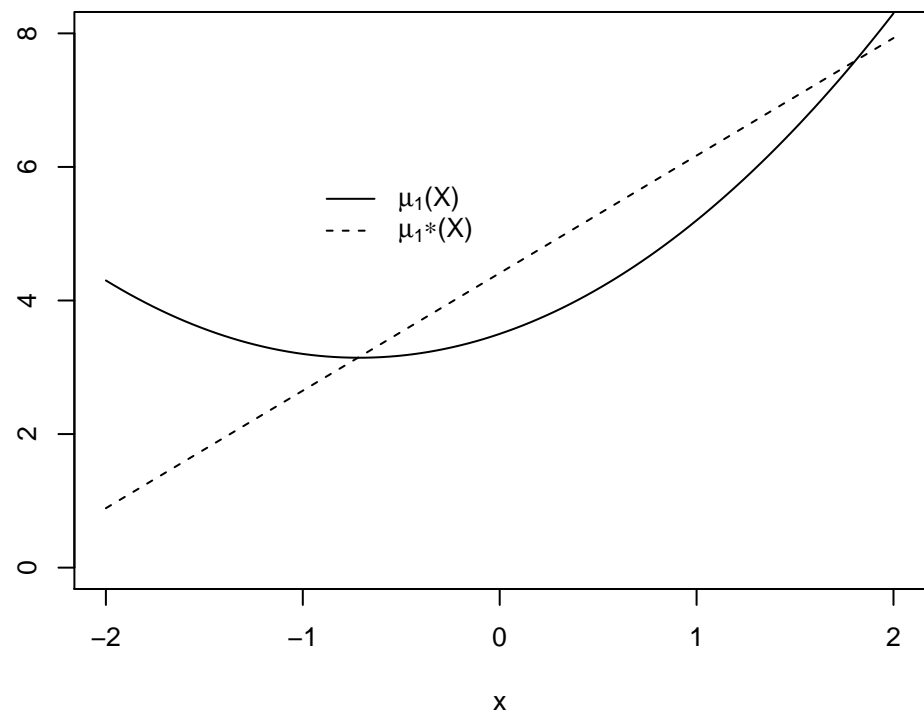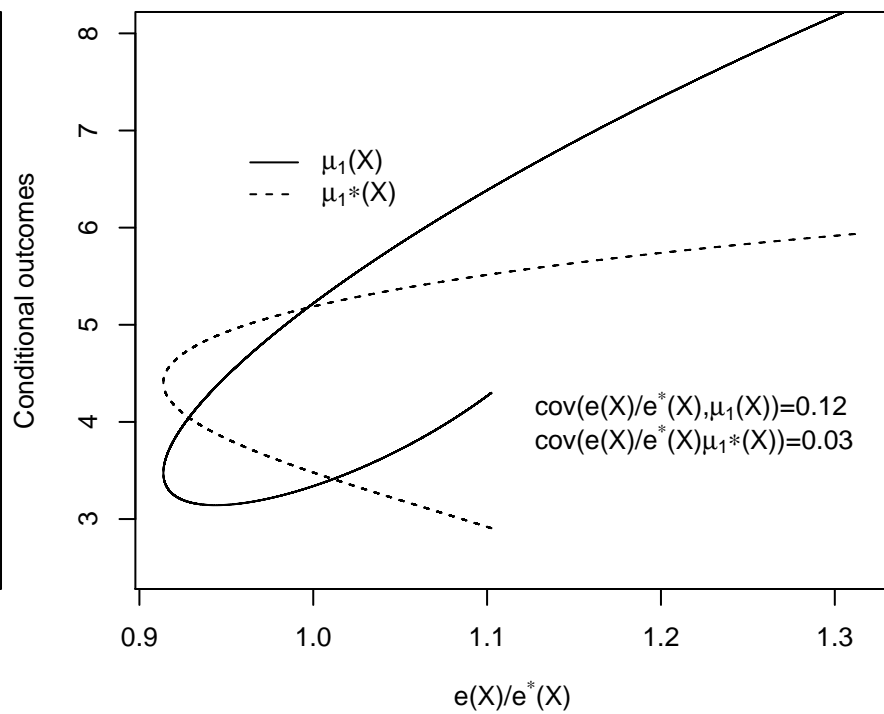

Supplement: Supplementary file 1 — Supporting Information. [file BIMJ-65-0-s001.zip › Code_and_Data/results/Figure1.pdf]

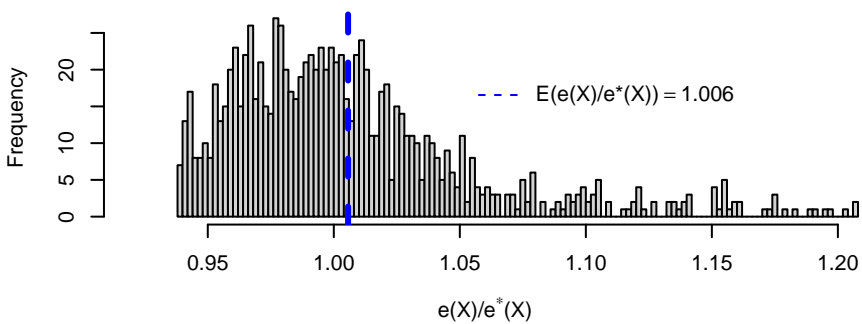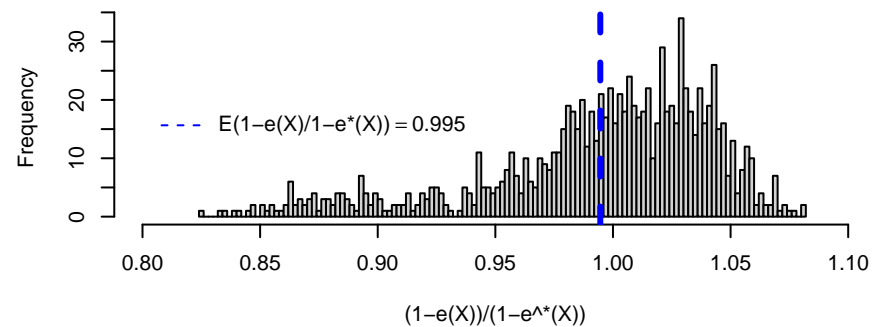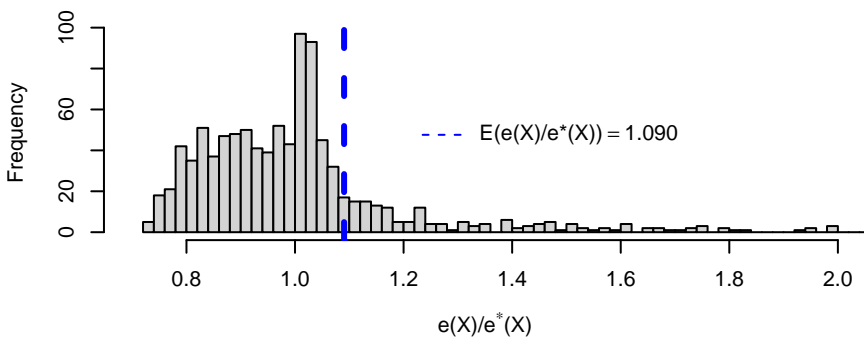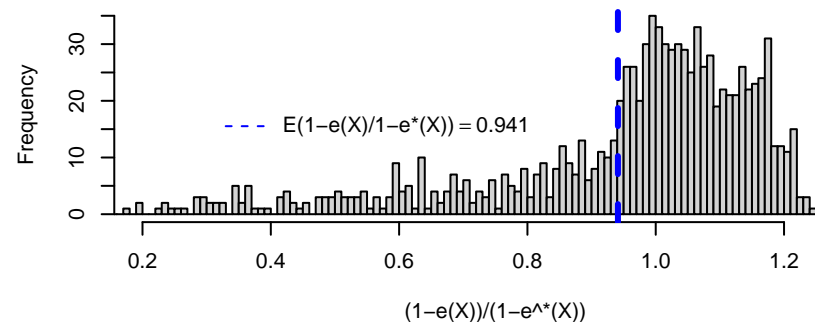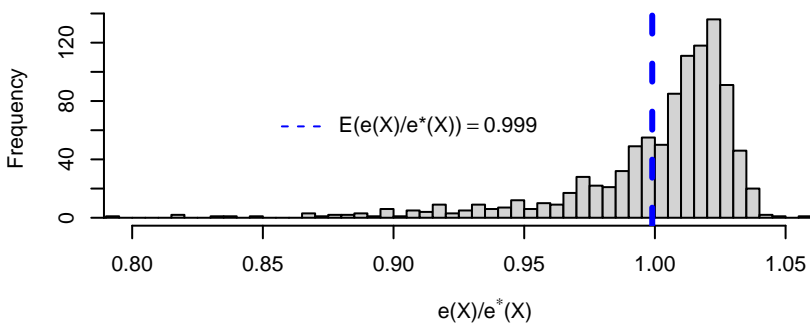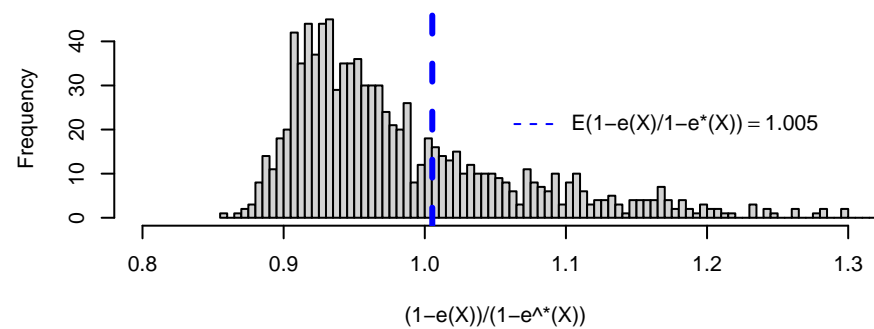

Supplement: Supplementary file 1 — Supporting Information. [file BIMJ-65-0-s001.zip › Code_and_Data/results/Figure2.pdf]

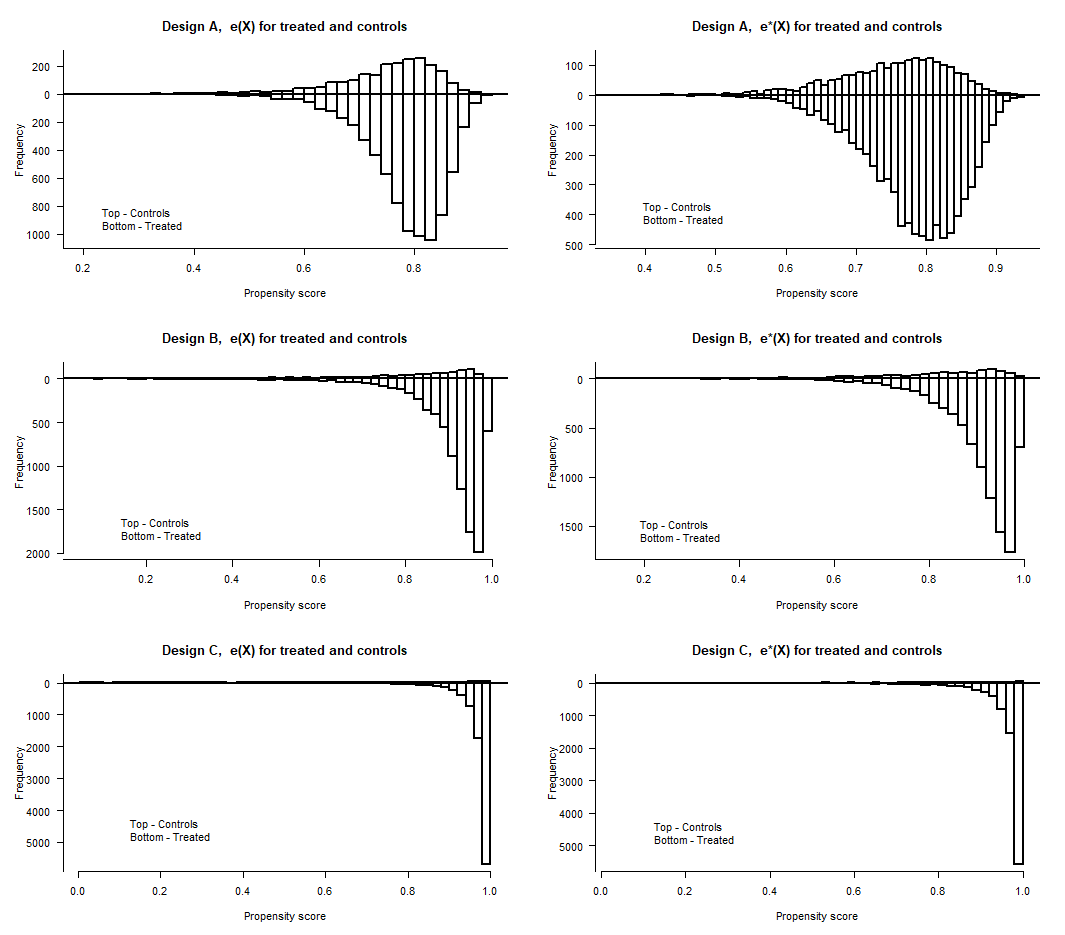

Supplement: Supplementary file 1 — Supporting Information. [file BIMJ-65-0-s001.zip › Code_and_Data/results/Figure5.png]

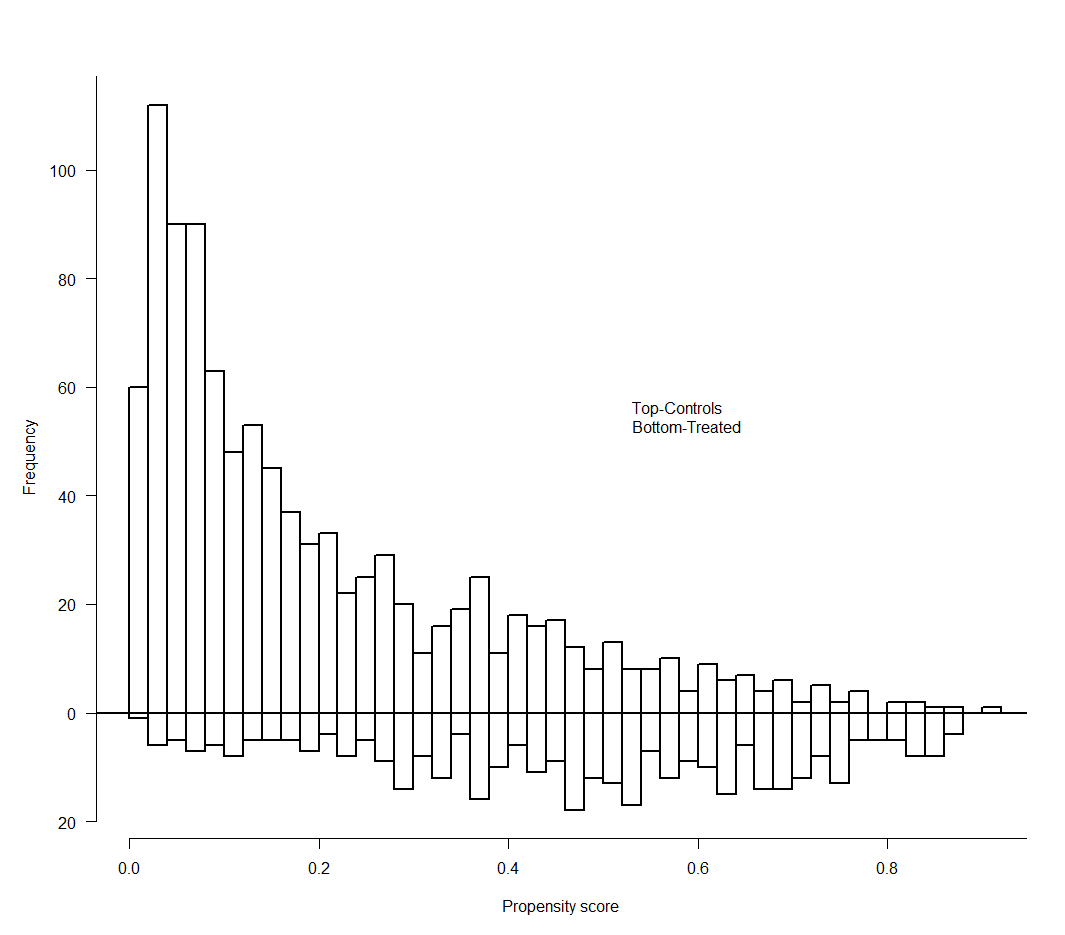

Supplement: Supplementary file 1 — Supporting Information. [file BIMJ-65-0-s001.zip › Code_and_Data/results/Figure6a.png]

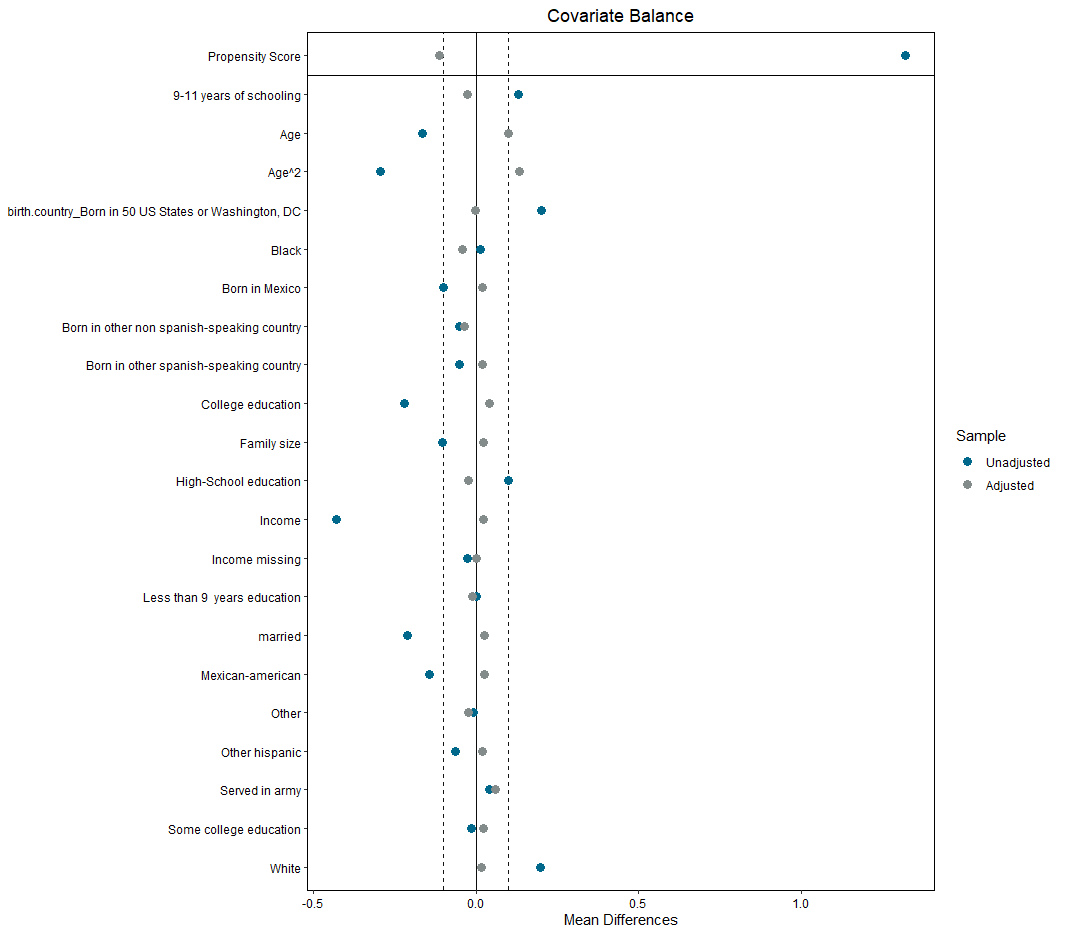

Supplement: Supplementary file 1 — Supporting Information. [file BIMJ-65-0-s001.zip › Code_and_Data/results/Figure6b.png]
